# Poly(ADP-ribosyl)ating enzymes cooperate to coordinate development

**DOI:** 10.1038/s41598-022-26530-2

**Published:** 2022-12-21

**Authors:** Guillaume Bordet, Iaroslava Karpova, Alexei V. Tulin

**Affiliations:** Department of Biomedical Sciences, School of Medicine and Health Sciences, University of North Dakota, 501 North Columbia Road, Stop 9061, Grand Forks, ND 58202 USA

**Keywords:** Computational biology and bioinformatics, Developmental biology, Genetics

## Abstract

The transcriptome is subject to rapid and massive changes during the transition between developmental stages. These changes require tight control to avoid the undesired reactivation of gene expression that is only important for previous developmental stages and, if unchecked during transition between developmental stages, could lead to anarchic proliferation and formation of malignant tumors. In this context, the involvement of chromatin factors is important since they can directly regulate the expression of multiple genes at the same time. Poly(ADP-ribose) enzymes, involved in several processes from DNA repair to transcription regulation, might play a role in this regulation. Here, we report that PARP-1 and PARG cooperate to temporally regulate the gene expression profile during the larval/pupa transition. PARP-1 and PARG are both essential in repressing the expression of genes coding for digestive enzymes and larval cuticle proteins, while PARG positively regulate the expression of defense response genes. These results suggest a cooperative coordination between PARP-1 and PARG that specifically maintains the integrity of expression profile between developmental stages.

## Introduction

**Figure 1:**
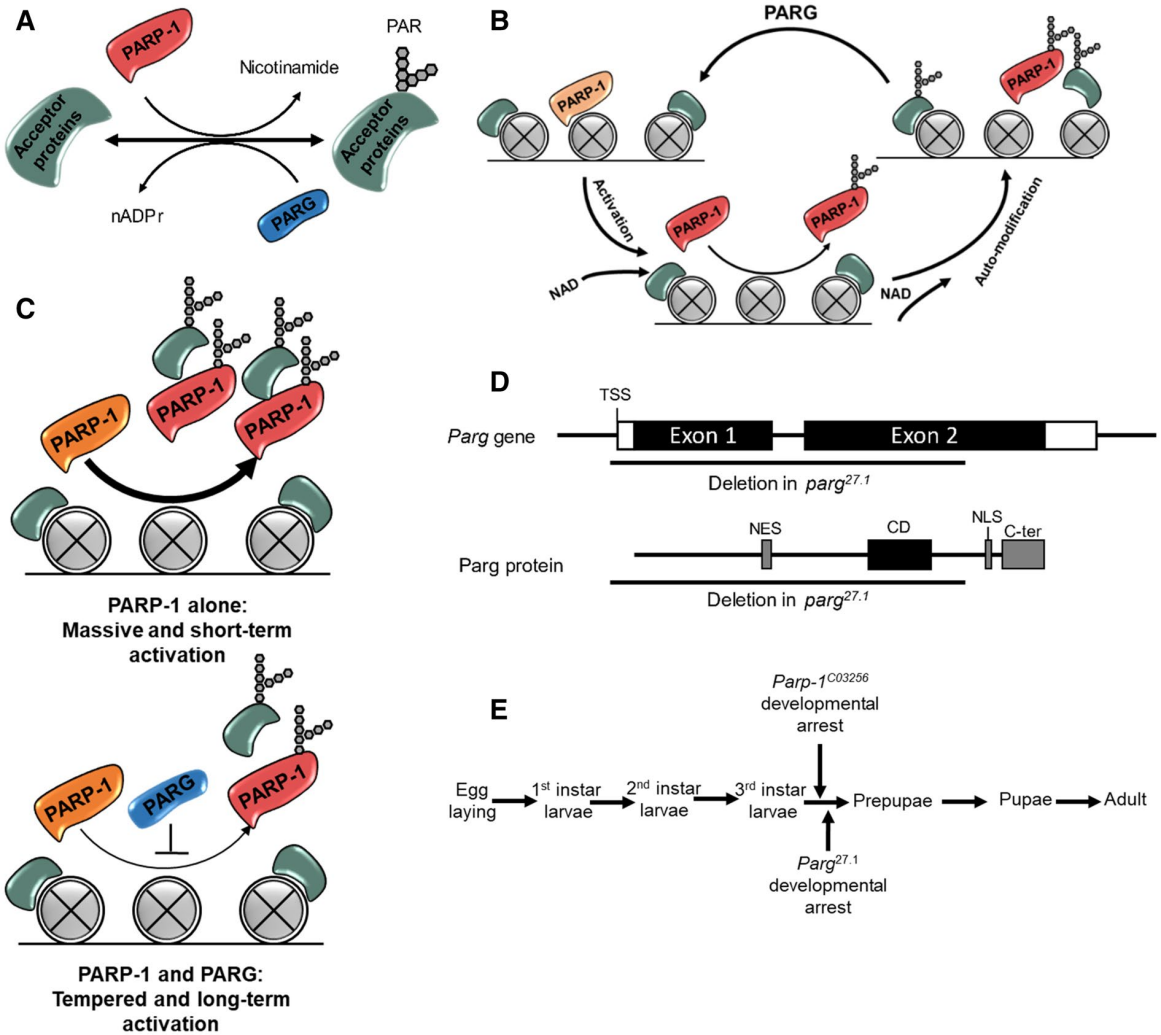
Poly(ADP-ribose) cycle and consequences of *Parp-1* and *Parg* mutants. (**A**) pADPr turnover. PAR: poly (ADP)ribose (**B**) PARG regulates PARP-1 activity. NAD: nicotinamide adenine dinucleotide. (**C**) The cooperation between PARP-1 and PARG. In absence of PARG, PARP-1 activity can only transient since its automodification leads to its dissociation with chromatin (Top panel). The presence of PARG temper PARP-1 activity by limiting PARP-1 automodification, allowing a long-term activation (lower panel). (**D**) the representation of Parg gene and the effect of *Parg*^*27.1*^ deletion. This deletion depletes the parg gene from just before the transcription start site (TSS) to the last third of exon 2, which includes the PARG catalytic domain (CD). (**E**) Drosophila life cycle and time point representing developmental arrest observed in *Parg*^*27.1*^ or *Parp-1*^*C03256*^ mutants.

The correct transition between two developmental stages often requires rapid changes in transcription programs, which, in turn, involves the activation of chromatin factors able to change chromatin structure to regulate the expression of a large number of genes^1^. These chromatin factors require strict control over their expression and activity since a defect in their regulation may lead to massive changes in the expression profile, including reactivation of developmental genes during later stages that might lead to anarchic proliferation and formation of malignant tumors^2^. Poly(ADP-ribose) polymerases (PARPs) are a family of enzymes involved in the poly(ADP-ribosyl)ation of acceptor nuclear proteins^3^. This post-translational modification add a strong negative charge to the acceptor proteins, leading to their dissociation from DNA and regulating their activity^4^ (Fig. 1A). The role of the PARP family, initially thought to be limited to DNA repair^3^, was progressively found to be important in many other biological processes, including chromatin compaction and loosening, as well as regulation of gene expression^5–13^. PARP targets are mainly chromatin proteins, including histones but also nucleic acids^14^. The main counterpart of PARP is poly(ADP-ribose) glycohydrolase (PARG), the main enzyme responsible for the removal of poly(ADP-ribose) (pADPr) moieties from acceptor proteins^15–17^. However, while PARG plays the role of the antagonist in the PARP family as the main remover of pADPr, PARP proteins are themselves targets of automodification, which can inhibit PARP DNA binding, protein–protein interactions, and ADP-ribosyl transferase activity, ultimately inactivating the protein^3,18^. PARP proteins poly(ADP-ribosyl)ate surrounding acceptor proteins and themselves, leading to the dissociation of all modified proteins from DNA, including PARP proteins themselves (Fig. 1B)^9^. Interestingly, histone proteins stay associated to chromatin even after poly(ADP-ribosyl)ation^19^. In this context, PARP activity can only be transient since automodification will prevent the interaction with chromatin (Fig. 1C, top panel). Then, the presence of PARG can help to maintain a long-term PARP activity by removing pADPr from PARP that can interact again with chromatin (Fig. 1C, lower panel). This suggests that the role of PARG is more subtle than being a PARP antagonist. This synergy between PARP-1 and PARG has been discussed^5,16^ but little is known about how their activity is regulated. *Drosophila melanogaster* is a good model to study the regulation of pADPr since the majority of the pADPr are metabolized by a single PARP protein (PARP-1) while the majority of pADPr is removed by PARG protein^17^. PARP-1 and PARG are mainly expressed at the beginning and the end of embryonic development (Fig.S1). Their expression is low during first and second instar larvae stage, but slightly increased before L3 larva puff stage 7–9, the last stage before pupation. *Parp-1*^*C03256*^ (hereafter termed as *Parp-1* mutant) is an hypomorphic version of *Parp-1* produced by the insertion of a pBac element in the fourth exon of *Parp-1*, resulting in expression of a truncated version of PARP-1 that presents strongly decreased activity^9^. *Parg*^*27.1*^ (hereafter termed as *Parg* mutant) is a deletion in the *Parg* gene triggered by a P-element excision, encompassing the transcription start site (TSS) to the end of the catalytic domain (Fig. 1D), leading to a complete depletion of *Parg* expression^15^. Theses mutants are maintained in a heterozygote state since homozygote mutants undergo developmental arrest at the end of the third instar larvae stage (L3 larva puff stage 7–9) and die before prepupa (Fig. 1E) ^9,15^, suggesting that both PARP-1 and PARG are important during this stage. The development from embryo to the end of third instar larvae is morphologically and temporally similar in *Parg* and *Parp-1* mutant than in wild type flies. Moreover, PARP-1 and PARG have been shown to play an important role earlier in development, but homozygote mutants are able to survive owing to a maternal loading of Parp-1 and Parg mRNA^20,21^. Interestingly, in some specific conditions, *Parg* mutants can produce sterile adults affected by progressive neurodegeneration^15^, suggesting that PARG is important in a broader developmental context all along the life of the flies.

Here we report that the absence of PARG leads to changes in gene expression profile similar to those in PARP-1 hypomorph context, suggesting that they play a similar role in the regulation of transcription. We also found that most upregulated differentially expressed genes (DEGs) in the absence of PARG or PARP-1 are developmental genes that should, in fact, be downregulated before the transition to the prepupal stage. These genes are involved in the formation of the larval cuticle, digestive enzymes, and the formation of primary metabolites. These results suggest that PARP-1 and PARG cooperate to regulate the expression of larval developmental genes before the transition to pupa.

## Results

### The absence of PARG affects the transcription profile at the end of third instar larvae

**Figure 2:**
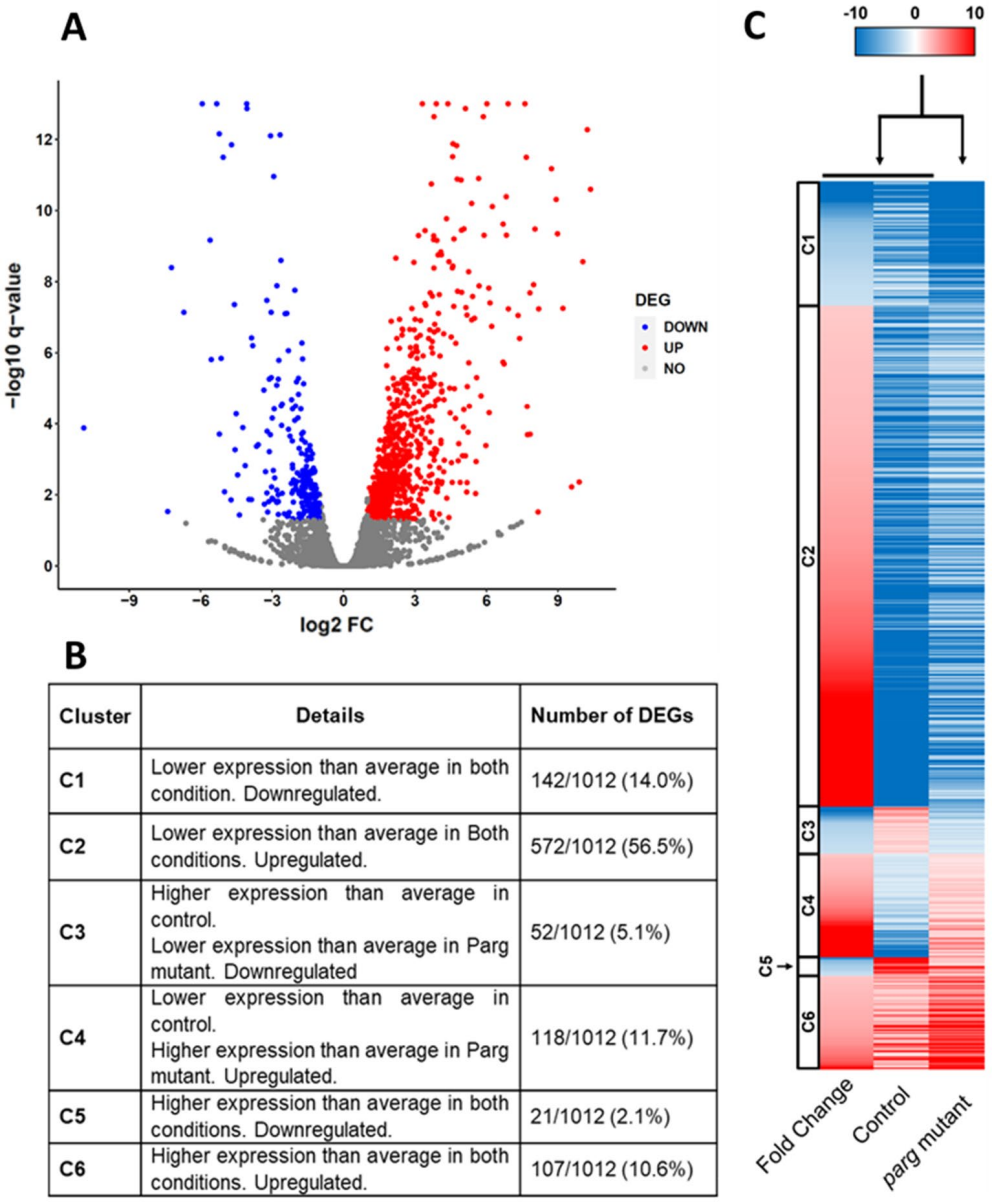
Expression profile is affected in the absence of PARG. (**A**) Volcano plot representing the fold change (FC) between *Parg* mutant and control larvae on the x-axis and the FDR-corrected p-value (q-value) of the y-axis. Significant number of downregulated genes (downregulated DEGs) are represented in blue, while a significant number of upregulated genes (upregulated DEGs) are represented in red. Genes without significant changes are represented in gray. DEG: differentially expressed genes. (**B**) Table representing the composition of the different clusters presented in Fig. 1C. Odd numbers include downregulated DEGs, while even numbers include upregulated DEGs. (**C**) Heatmap of DEGs. The blue shading of the fold change column represents downregulated DEGs, while the red shading represents upregulated DEGs. The control and *Parg* mutant columns represent the expression value of DEGs normalized to the average expression of all genes expressed in control or *Parg* mutant, respectively. For control and Parg mutant columns, blue shading represents DEGs with lower than average expression, while red shading represents DEGs with higher than average expression. Heatmap was generated with Excel 2016, available at this link: https://www.microsoft.com/en-us/microsoft-365/excel.

To compare the expression profile of third instar larvae in the presence or absence of PARG, we used a line expressing *Parg*^*27.1*^ mutant (hereinafter termed as *Parg* mutant) and compared it to a control line expressing a similar genetic background (hereinafter termed as control) since these two lines share the same genetic background. We selected larvae at the end of L3 larva puff stage 7–9 before *Parg* mutant developmental arrest. The expression profile was analyzed based on RNA-seq of three different biological replicates. Differentially expressed genes (DEGs) were identified based on the following criteria: fold change (FC) higher than 2, False Discovery Rate (FDR)-corrected p-value (q-value) lower than 0.05 based on two-tailed *t*-test and Benjamini–Hochberg correction, and an average reads per kilobase million (RPKM) higher than 1 in at least one condition (*Parg* mutant or control). Using these criteria, among 13,601 genes present in *Drosophila melanogaster,* we identified 1012 genes differentially expressed between *Parg* mutant and control larvae, among which 215 are downregulated in *Parg* mutant compared to control (hereinafter termed as downregulated DEGs), while 797 are upregulated in *Parg* mutant (hereinafter termed as upregulated DEGs) (Fig. 2A). Gene expression fold change is wide, from − 1919.17 for gene *Cg46276* with “unknown function” to 1320.86 for gene *Cpr47Eg* coding for a larval cuticular protein. However, 819 of the DEGs (80.9%) present a fold change between − 10 and 10 (Fig. 2A). As expected, *Parg* is a highly downregulated DEG with a fold change of − 40. To confirm RNA-seq results, we selected seven genes that present different fold changes: *Cg14850*; *Mtnc* and *Parg*, representing downregulated DEGs; *Nfat*, downregulated, but not significantly; and *Aest7*, *Cyp6w1* and *Ninad*, representing upregulated DEGs. We performed qPCR for all these targets with new samples prepared as previously noted. The qPCR results corroborate those found from RNA-seq (Fig.S2). Furthermore, the correlation between fold change in qPCR and RNA-seq is 0.995, suggesting that our RNA-seq results are consistent.

To sort the different DEGs based on their expression level (RPKM), we split them into six different clusters (Fig. 2B–C) based on their expression in *Parg* mutant and control groups and normalized by the average RPKM of all genes. This normalization to average expression allows us to temper high fold change when expression is low, to avoid artifact. Cluster 1 (C1) includes 142 DEGs downregulated in *Parg* mutant and expressed at levels lower than average in *Parg* mutant and control groups. Cluster 2 (C2) includes 572 DEGs upregulated in *Parg* mutant and expressed at levels lower than average in both conditions. Cluster 3 (C3) includes 52 DEGs downregulated in *Parg* mutant and expressed at levels lower than average in *Parg* mutant, but higher than average in control. Cluster 4 (C4) includes 118 DEGs upregulated in *Parg* mutant and expressed at levels higher than average in *Parg* mutant, but lower than average in control. Cluster 5 (C5) includes 21 DEGs downregulated in *Parg* mutant and expressed at levels higher than average in both conditions. Cluster 6 (C6) includes 107 DEGs upregulated in *Parg* mutant and expressed at levels higher than average in *Parg* mutant and control groups. This normalization allows us to sort the DEGs based on their expression level and give us a first idea of how the expression profile is affected in absence of PARG (Fig. 2C). Around 66% of downregulated DEGs and upregulated DEGs present a lower expression than average in both conditions (cluster 1 and 2) (Fig. 2C). Furthermore, 149 out of the 192 DEGs that present a fold change lower than − 10 or higher than 10 belong to cluster 1 and 2 (77.6%), suggesting that the high fold change we observed among DEGs results from lower gene expression than average. Interestingly, the average RPKM value of all expressed genes is similar for both conditions (47.9 for control and 50.1 for *Parg* mutant), while the average RPKM value for all DEGs is different (66.2 for control and 143.4 for *Parg* mutant), suggesting that in a specific set of genes present a higher expression in absence of PARG than in control group. In summary, these results indicate that the absence of PARG leads to the upregulation of a specific set of genes, suggesting, in turn, that PARG play a role in the repression of gene expression during third instar larvae stage rather than in their activation. However, since changes in the expression of low-expressed gene can have severe consequences (e.g. transcription factors) this normalization was done only for clustering purpose and will not be used in the rest of the manuscript.

### Expression profiles correlate between *Parg* and *Parp-1* mutants

**Figure 3:**
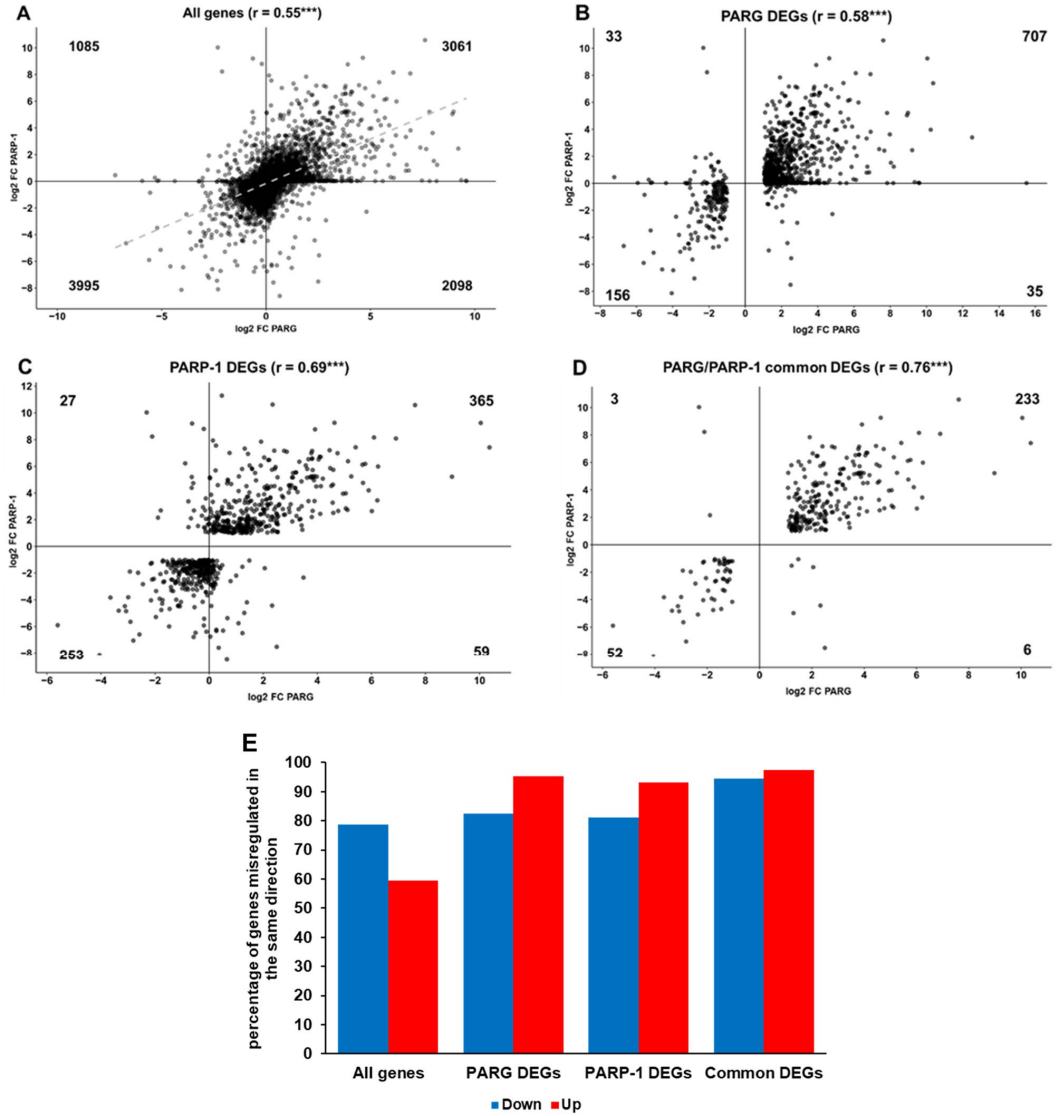
*Parp-1* and *Parg* mutants present similar changes in their expression profiles. (**A**-**D**) Scatter plots representing the expression of (**A**) 10 239 genes expressed in both *Parp-1* and *Parg* mutants, (**B**) 931 PARG DEGs expressed in *Parp-1* mutant, (**C**) 704 PARP-1 DEGs expressed in *Parg* mutant, and (**D**) 294 PARP-1/PARG common DEGs. X-axis shows a log2 scale of Fold Change (FC) in the absence of PARG compared to control, while Y-axis shows a log2 scale of FC in the absence of PARP-1 compared to control. The number present in each quadrant corresponds to the number of genes present in this quadrant. The r number is the Pearson correlation coefficient. The *p*-value is calculated based on the Pearson correlation coefficient and the t-distribution with n-2 degrees of freedom. ***p < 0.01. (**E**) Summary of the data represented in (**A**–**D**). Genes downregulated in both mutants are represented in blue, while genes upregulated in both mutants are represented in red. This graph is a comparison between X- and Y-axis presented in (**A**–**D**). For example, the red bar in the “All genes” category means that 59.3% of the genes expressed in both mutants and upregulated in the *Parg* mutant are also upregulated in the *Parp-1* mutant.

We previously reported that PARP-1 and PARG proteins physically interact in *Drosophila*^22^. Moreover, these two proteins are co-localized in *Drosophila* polytene chromosomes chromatin (Fig.S3). We previously published microarray data comparing expression profile between wild type and *Parp-1* mutant during L3 larva puff stage 7–9^23^ and identified 703 DEGs. We then compared changes in the expression profile between both mutants. Since microarray results are limited to the probes present on the chip, we were able to compare the expression profile of only 10,239 genes between *Parp-1* and *Parg* mutant. Their expression in the absence of PARP-1 strongly correlates with their expression in the absence of PARG (r = 0.55) (Fig. 3A). This correlation can also be observed among PARG DEGs (Fig. 3B, r = 0.58) and PARP-1 DEGs (Fig. 3C, r = 0.69). We identified 294 genes significantly misregulated in both mutants (Fig. 3D), but still presenting strong correlation (r = 0.76). Among them, 234 (79.2%) are upregulated in both mutants. Overall, more than 80% of downregulated DEGs in one mutant are downregulated in the other mutant, while more than 95% of upregulated DEGs in one mutant are upregulated in the other mutant (Fig. 3E). Furthermore, *Parg* expression is not affected in *Parp-1* mutant while *Parp-1* expression is not affected in *Parg* mutant, suggesting that this correlation is not due to change in the expression of one enzyme in absence of the other. Taken together, these results suggest that PARG and PARP-1 are involved in parallel in the regulation of expression of a common set of target genes.

### PARG and PARP-1 regulate the expression of developmental genes

**Figure 4:**
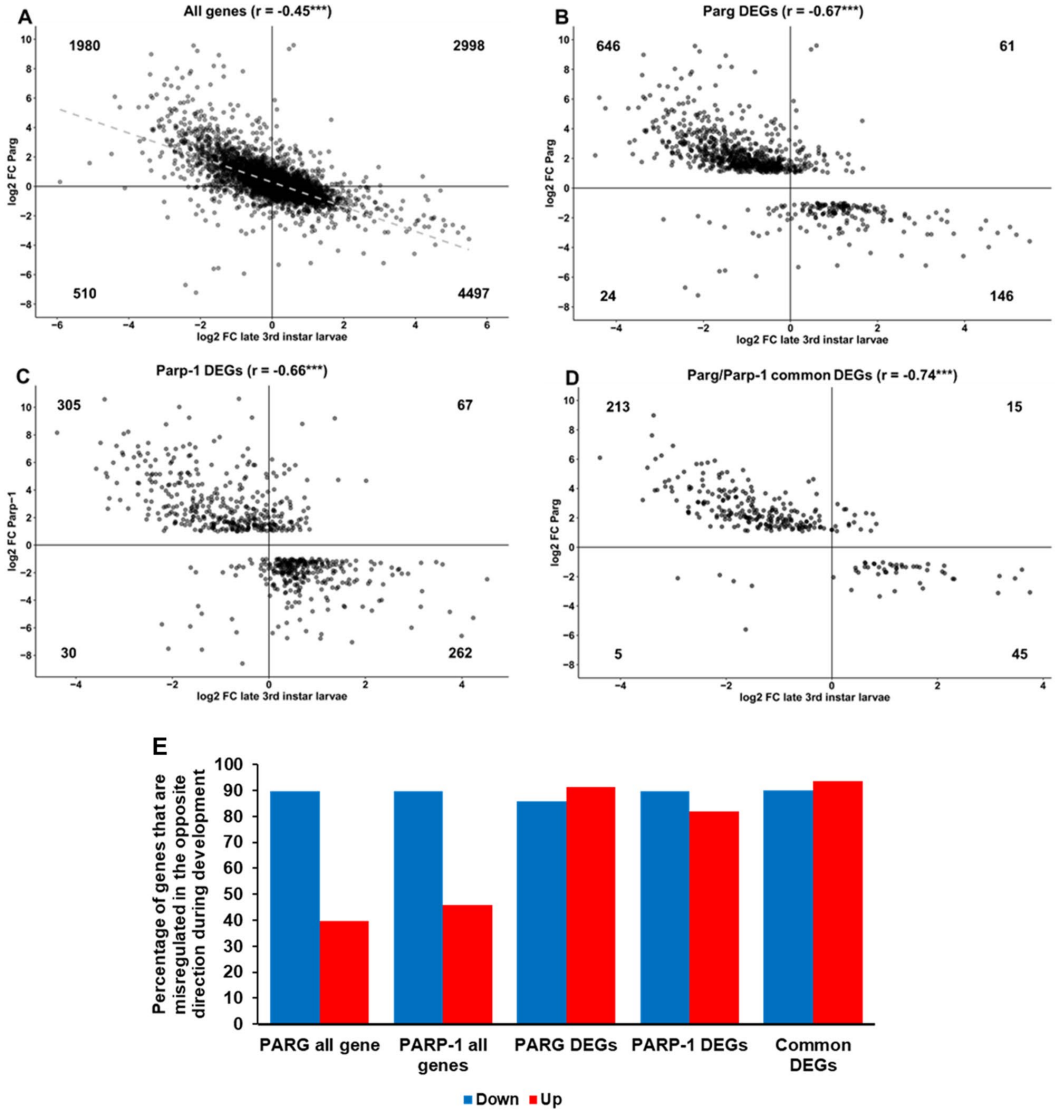
The absence of PARP-1 or PARG affects the expression of developmental genes. (**A**-**D**) Scatter plots representing the expression of (**A**) 9 985 genes expressed in *Parg* mutant and during L3 larva puff stage 3–6 and 7–9, (**B**) 877 PARG DEGs expressed in *Parg* mutant and during L3 larva puff stage 3–6 and 7–9, (**C**) 664 PARP-1 DEGs expressed in *Parg* mutant and during L3 larva puff stage 3–6 and 7–9, and (**D**) 278 PARP-1/PARG common DEGs expressed in *Parg* mutant and during L3 larva puff stage 3–6 and 7–9. X-axis shows a log2 scale of the Fold Change (FC) between L3 larva puff stage 3–7 and 7–9, while Y-axis shows a log2 scale of the FC in the absence of PARG compared to control for (**A**,** B** and** D**), or in the absence of PARP-1 for (**C**). The number present in each quadrant corresponds to the number of genes present in this quadrant. The r number is the Pearson correlation coefficient. The p-value is calculated based on the Pearson correlation coefficient and the t-distribution with n-2 degrees of freedom. ***p < 0.01. (**E**) Summary of the data represented in **A**–**D**. Genes downregulated in mutants and upregulated during L3 larva puff stage 7–9 are represented in blue, while genes upregulated in mutants and downregulated during L3 larva puff stage 7–9 are represented in red. This graph is a comparison between X- and Y-axis presented in **A**–**D**. For example, the red bar in the “All genes” category means that 39.8% of the genes upregulated in the *Parg* mutant should be downregulated during L3 larva puff stage 7–9.

Since both *Parg* and *Parp-1* mutants present developmental arrest before the end of third instar larvae puff stage 7–9, we wanted to see how the expression of PARG and PARP-1 DEGs varies during this stage. To make this determination, we used RNA-seq data published in Graveley et. al.^24^ to compare the expression profile between L3 larvae puff stage 7–9 and the previous developmental stage L3 larvae puff stage 3–6. Interestingly, we found a significant moderate anti-correlation (r = − 0.45) between the changes in the expression profile in the absence of PARG and the changes that should occur at this developmental stage (Fig. 4A). Furthermore, this anti-correlation is even stronger for PARG DEGs (r = − 0.67) (Fig. 4B), having 646 DEGs out of 707 (91.4%) that should be downregulated during L3 larva puff stage 7–9, but are upregulated in the absence of PARG. We also found a similar anti-correlation among PARP-1 DEGs (r = − 0.66) (Fig. 4C). The strongest anti-correlation we observed was among PARP-1/PARG common DEGs (r = − 0.74) where 213 out of 228 DEGs (93.4%) that should be downregulated during L3 larva puff stage 7–9 are, instead, upregulated in both mutants (Fig. 4D). Overall, then, the expression of most DEGs goes in an opposing direction in the absence of either PARG or PARP-1, rather than the direction they should follow in normal development (Fig. 4E) for both down- and upregulated DEGs. Interestingly, only 39.8% and 45.8% of the genes upregulated in *Parg* or *Parp-1* mutant respectively (significantly or not) are supposed to be downregulated during L3 larva puff stage 7–9, while 91.4% of PARG upregulated DEGs and 82.0% of PARP-1 upregulated DEGs should be downregulated during this developmental stage (Fig. 4E), suggesting that PARG and PARP-1 are involved in the regulation of a specific set of developmental genes during this stage rather than in the global regulation of gene expression. Taken together, these results suggest that the absence of PARG or PARP-1 affects the expression of developmental genes.

### PARP-1 and PARG upregulated DEGs are involved in developmental functions

**Figure 5:**
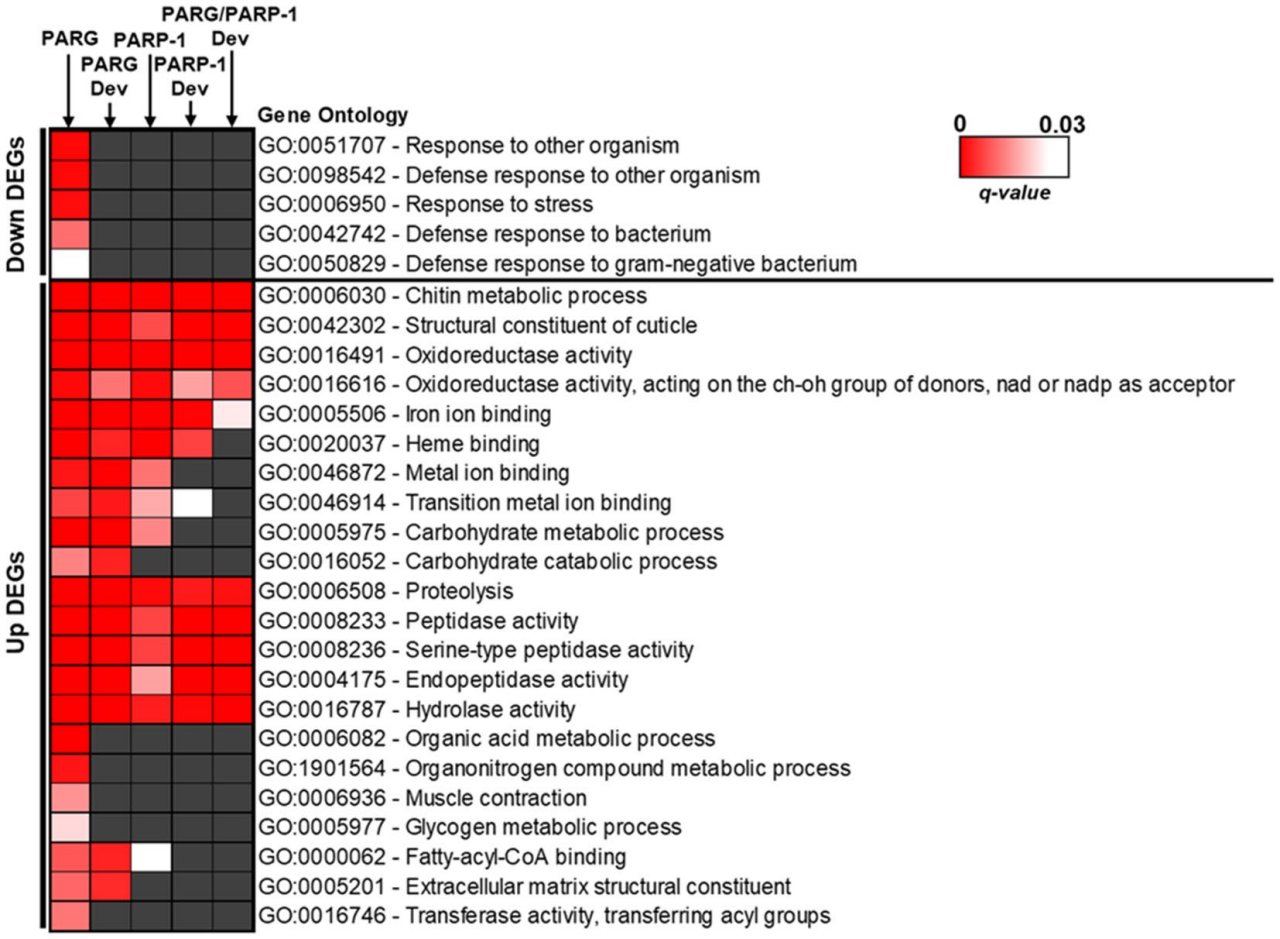
PARG and PARP-1 DEGs are involved in specific functions. Overview of the main Gene Ontology-terms (GO-terms) overrepresented among DEGs. The upper part corresponds to the downregulated DEGs, while the lower part corresponds to upregulated DEGs. The left part corresponds to a heatmap of the FDR-corrected *p*-value (q-value) associated with the different GO-terms. The red-to-white shaded tiles correspond to GO-terms that are significantly overrepresented among DEGs, while dark gray tiles correspond to GO-terms not significantly enriched. The left column, titled “PARG”, includes the 215 PARG downregulated DEGS and the 797 upregulated DEGs. The second column, titled “PARG Dev”, is a subtype of the first column that included the 146 PARG downregulated DEGs that should be upregulated through development and the 646 PARG upregulated DEGs that should be downregulated through development. The third column, titled “PARP-1”, includes the 311 PARP-1 downregulated DEGs and the 392 upregulated DEGs. The fourth column, titled “PARP-1 Dev”, is a subtype of the third column that includes the 262 PARP-1 downregulated DEGs that should be upregulated through development and the 305 upregulated DEGs that should be downregulated through development. The fifth column, tilted “PARG/PARP-1 Dev”, includes the 45 PARG/PARP-1 common downregulated DEGs that should be upregulated through development and the 213 common upregulated DEGs that should be downregulated through development. q-value is a Benjamini–Hochberg corrected p-values based on multiple tests performed using the STRING database ^25^. Heatmap was generated with Excel 2016, available at this link: https://www.microsoft.com/en-us/microsoft-365/excel.

We investigated the functions of PARG and PARP-1 DEGs using the STRING database^25^ to see if they are involved in developmental functions. First, we analyzed the list of PARG downregulated DEGs. We found only 5 gene ontology terms (GO) overrepresented among downregulated DEGs, and all of them are involved in defense response (Fig. 5). Overall, this overrepresentation applies to only 38 genes (17.7%) among the 215 downregulated genes, suggesting that most share no common functions. Furthermore, we analyzed the gene ontology information associated with each downregulated DEG available on Flybase^26^. Among the 215 downregulated DEGs, we found that 53 (24.7%) have no associated molecular function or biological process gene ontology, leaving 124 DEGs (57.7%) that have known function, but not shared with other DEGs. This result suggests that PARG is not involved in the activation of genes associated with a specific function. Interestingly, these defense response GO-terms are not overrepresented among PARG downregulated DEGs that are supposed to be upregulated during development (Fig. 5, Table.S1), suggesting that the involvement of PARG in the regulation of defense response genes is independent of developmental changes. Furthermore, we did not find an overrepresentation of defense response GO-terms among PARP-1 DEGs or among PARP-1 DEGs and PARP-1/PARG sharing common functions that should be upregulated during development (Fig. 5), suggesting that this involvement in the regulation of defense response genes is PARG-specific.

Then, we analyzed the function of PARG upregulated DEGs. We found 53 GO-terms that are overrepresented among upregulated DEGs (Fig. 5). The top functions we found overrepresented are related to chitin synthesis and cuticle development, including 91 genes upregulated in absence of PARG. We also identified that 179 of the upregulated DEGs present hydrolase activity, which is one the most represented categories. Among these 179 genes, 95 (53.1%) are involved in proteolysis. We identified that 32 of the upregulated DEGs are involved in heme binding and that among these, 29 are members of the cytochrome family. We also identified eight genes involved in muscle contraction. Overall, 469 (58.8%) of the 797 upregulated DEGs belong to at least one of the overrepresented GO terms, while 168 (21.1%) have no associated molecular function or biological process GO-term, leaving 160 genes (20.1%) that have known function not shared with other DEGs. This suggests that PARG is involved in the repression of genes associated with a specific function. However, among all the other tested groups of DEGs, only those involved in chitin metabolic process, proteolysis and oxidoreductase activities are overrepresented, suggesting that the involvement of PARG in other functions, such as muscle contraction, is independent of developmental changes.

We identified 31 genes involved in cuticle formation that are upregulated in the absence of PARG that should be downregulated at the end of L3 larva puff stage 7–9 (Table.S2). All of them are expressed during larval stages, and their expression strongly decreases before white prepupa stage^24^ (Table.S2), suggesting that they are involved in cuticle formation during larval stage, but not needed for the transition to pupal stage. Next, to determine in more detail the role of the 66 genes involved in proteolysis, we analyzed their expression profile using Flymine tools^27^ and found that 53 are expressed in larval midgut (80.3%) (Fig.S4). Furthermore, among these 53 genes, 47 (88.7%) have been reported to code for digestive enzymes^28^ (Table.S3). It is well known that larvae start starvation after reaching the critical weight required to start pupation^29^. The L3 larva puff stage 7–9 is also called “clear gut” stage since the gut is empty. Digestive enzymes are not required at this stage, explaining why they are downregulated. Finally, we analyzed in more detail the role of the 52 genes involved in oxido-reduction using STRING (Fig.S5) and found that they are involved in different functions, including generation of precursor metabolites and metabolic processes involving glycogen, NAD, lipid, alcohol and carnitine. However, among the PARG and/or PARP-1 upregulated DEGs downregulated during development, we found only GO-terms involved in the generation of precursor metabolites and metabolic processes of alcohol and carnitine, suggesting that the role of PARG in glycogen, lipid and NAD metabolic processes is independent of developmental changes. Taken together, these results suggest that PARG and PARP-1 are involved in the repression of larval developmental genes before transition to pupa and whose expression is not required during pupal stage.

### PARG DEGs are organized in clusters

During our analysis, we found that several PARG upregulated DEGs are organized in clusters of genes sharing the same functions (Table.S4). These clusters are mainly genes involved in chitin formation or digestive enzymes already known to form clusters^30,31^. However, it is interesting to note that almost all of them are significantly misregulated in *Parg* mutant, raising the possibility that PARG and PARP-1 regulates them together. To better characterize that speculation, we measured the distance between two DEGs and compared their distribution to a control group wherein gene positions are randomized (Fig.S6A–B). Overall, we found the DEGs loci to be genetically closer from each other than expected, given the randomized positioning, suggesting that PARG DEGs are organized mainly in clusters.

## Discussion

Here, we reported that the absence of PARG affects the gene expression profile during L3 larva puff stage 7–9. The list of genes affected by the absence of PARG overlaps strongly with the list of genes affected in the absence of PARP-1^23^ and common targets are in a large majority upregulated in both mutants, suggesting that PARP-1 and PARG cooperatively repress the expression of common targets in the same pathway. This overlap is consistent with the changes observed in MCF-7 human cells when *Parp-1* or *Parg* is knocked down^32^. The absence of PARG or PARP-1 leads to a developmental arrest before transition to pupation, suggesting that they are both important for the transition. Furthermore, the development before this arrest in similar with control development, suggesting that the presence of PARP-1 and PARG is critical for this transition but not for larval development before. This is consistent with our previous report that showed that PARP-1 is highly active during the L3 larva puff stage 7–9, whereas its activity remains low during the other larval stages^16^. Furthermore, we reported that PARP-1 activity is sensitive to the presence of 20-hydroxyecdysone^16^ and that one peak of ecdysone occurs at the end of third larval stage during the transition to pupa^33^. This raises the possibility that PARP-1 activation during L3 larva puff stage 7–9 is triggered by the ecdysone peak. Here, we reported that the absence of PARG or PARP-1 leads to the upregulation of genes that should be downregulated before pupation, including most associated with digestive enzymes, larval cuticle constituents and proteins involved in the generation of metabolite precursors, suggesting that PARP-1 and PARG are involved in their repression. PARP-1 has been reported to play a role in gene activation mainly^10,11,34–36^. However, PARP-1 can also be involved in the repression of transcription, such as PD-L1 that is repressed through STAT3 poly(ADP-ribosyl)ation in human cancer cells^37^, or retrotransposon repressed by PARP-1 in *Drosophila*^9^. PARP-1 is also involved in the direct repression of FoxO1 transcription factor^38^, and, interestingly, this repression is independent of its polymerase activity. Another possible scenario would be that PARP-1 and PARG are essential for ecdysone activation. This scenario could explain that both mutants fail to repress the expression of larval genes before the transition to pupa. However, this scenario is unlikely for several reasons. First, we previously reported that PARP-1 can be activated by 20-hydroxyecdysone^16^, suggesting that PARP-1 act downstream of the ecdysone pathway (Table.S5). Second, the expression of the enzymes responsible for the synthesis of 20-hydroxyecdysone^39^ is not affected in *Parg* or *Parp-1* mutants. Third, the expression of ecdysone early-response gene such as glue genes^40^ is still initiated in both *Parg* or *Parp-1* mutants. Fourth, the expression of ecdysone-response genes that start their expression around L3 larvae puff stage 7–9 is still initiated (Table.S5). Fifth, at the end of the third instar larvae the larvae change their behavior and start a wandering phase (they leave the food to find a pupation spot). This behavior is strongly suspected to be triggered by the ecdysone pathway^41^. This change of behavior is still present in both mutants. All together these elements suggests that the ecdysone pathway is still activated in absence of PARP-1 or PARG and raise the possibility that PARP-1 is activated by the ecdysone peak or by an event immediately downstream at the end of third instar larvae, while PARG is activated by the presence of pADPr to limit PARP-1 automodification, leading to a PARP-1 long-term activation (Fig. 1C). This cooperation between the two enzymes leads to the repression of developmental genes, the expression of which is no longer needed in the new stage of development.

It is likely that PARP-1 and PARG regulate gene expression in parallel with other transcription factors. For instance, we found that a “GATAAG” motif, highly similar to a GATA motif, is enriched at the promoter of the genes upregulated in *Parg* or *Parp-1* mutants (66.6%), while this motif is not enriched among downregulated genes (Fig.S7A-B). This motif is present at two possible locations: just before the transcription start site (TSS) and around − 400 from the TSS. 65% of these genes have a single copy of this GATA motif, while 20% present two copies. To identify the proteins known to bind to a similar motif, we performed a TomTom analysis^42^ and identified 4 out of the 5 GATA factors reported in *Drosophila* (Fig.S7C): Serpent, Gatae, Panier, and Gatad^43^, suggesting that they might bind to PARG/PARP-1 upregulated DEGs and participate, as well, in their repression. GATA factors are mainly reported to play a role in gene activation, but they can also be involved in gene repression^44^. Since GATA factors are pioneer factors^45^, it is possible that they participate in recruiting PARP-1 for the repression of genes. Furthermore, GATA factors and PARP-1 has been shown in mice to physically interact and cooperate in the regulation of common targets^46^, PARP-1 acting as a co-factor of Gata factors. *Drosophila* Gata factors are mainly expressed during embryonic development and present a low expression during early larval stages (Fig.S8). However, the expression of all factors, except Gatae, increases slightly before L3 larva puff stage 7–9. All together, these elements suggest that GATA factors and PARP-1 might cooperate in the regulation of transcriptional changes observed during the larva/pupa transition.

The presence of genes involved defense response among PARG downregulated DEGs was unexpected. Furthermore, the involvement of PARG in their regulation is independent from developmental changes (Fig. 5). Poly(ADP-ribosyl)ating enzymes has been reported to regulated defense response genes in *Arabidopsis thaliana*^47^ and to regulate the antiviral response in mammals^48^. Then, these data suggest that PARG play such a role in *Drosophila* as well.

Here we described a cooperative developmental role of PARG and PARP-1 during the transition from larval to pupal stage that requires a change in the expression of several gene pathways. However, it is likely that this role of PARG and PARP-1 in the regulation of transcription profile is not restricted to the transition from larva to pupa. Our lab previously reported that the disruption of *Parg* or *Parp-1* during embryonic development leads to developmental arrest before hatching^20,21^ suggesting that they both play a role during embryonic development. It is possible that PARP-1 an PARG regulate the transcriptional changes during other developmental transition such as during embryonic development.

## Materials and methods

### Drosophila strains and genetics

Stocks obtained from the Bloomington Drosophila Stock Center (NIH P40OD018537) were used in this study. Flies were raised at 20 °C. The *Parp-1*^*C03256*^ strain was generated in a single pBac-element mutagenesis screen^49^. Precise excision of *Parp-1*^*C03256*^ was carried out using pBac transposase on CyO chromosome. Balancer chromosomes carrying Kr::GFP, i.e., TM3, Sb, P{w + , Kr-GFP4} and FM7i^50^, were used to identify heterozygous and homozygous *Parp-1*^*C03256*^. The *Parg*^*27.1*^ strain was obtained from^15^. A yellow white strain carrying the mutations *y*^*1*^*, w*^*1118*^ was used as a control. Since *Parg*^*27.1*^ is lethal at homozygote or hemizygote state, the stock was balanced with FM7iG balancer (Bloomington stock 4559).

### RNA-seq sample preparation and sequencing

This experiment was performed with three biological replicates for *Parg* mutant and control groups. *Parg* mutant larvae were obtained by crossing *Parg*^*27.1*^*/FM7iG* females with *FM7iG/Y* males. Twelve GFP-negative larvae were selected at L3 larvae puff stage 7–9 for each replicate. All GFP-negative larvae were males (*Parg*^*27.1*^*/Y*). Control larvae were generated by crossing *w*^*1118*^*, y*^*1*^*/FM7iG* females with *FM7iG/Y* males. Twelve GFP-negative larvae at L3 larva puff stage 7–9 stage were selected for each replicate. All GFP- negative larvae were males (*w*^*1118*^*, y*^*1*^*/Y*). Total RNA was purified from the whole organism using the QIAshredder column and RNeasy Mini kit (Qiagen, Valencia, CA). The quality of RNA was determined by Bioanalyzer with the RIN score ranging from 7 to 7.3 between samples. RNA library preparation and paired-read sequencing was performed by Novogene Corporation, Sacramento CA. mRNA was enriched based on a Poly(A) selection. Libraries were prepared using NEB’s Ultra II RNA library kit, and sequencing was performed on the NovaSeq 6000 system (Illumina, USA). Illumina FQ sequencing was analyzed with CLC Genomics Workbench, version 12.0. The reads were trimmed to remove adapters and mapped to the Dm6 *Drosophila melanogaster* genome using CLC’s default settings (mismatch cost = 2, insertion cost = 3, deletion cost = 3, length fraction = 0.8, similarity fraction = 0.8, auto-detect paired distances, maximum number of hits for a read = 10). Differential expression was calculated by comparing expression profiles between *Parg* mutant and control groups. Raw data were uploaded on GEO platform^51,52^ The data are available with the following link: https://www.ncbi.nlm.nih.gov/geo/query/acc.cgi?acc=GSE200499.

### Quantitative RT-PCR assays

This assay was performed in triplicate. Twelve third instar larvae were collected for *Parg* mutant and control groups. Total RNA was purified using the QIAshredder column and RNeasy kit (Qiagen). cDNA was obtained by reverse transcription using M-NLV reverse transcriptase (Invitrogen) using 6 µg of purified RNA for each sample. Real-time PCR assays were run using SYBR Green master mix (Bio-Rad) volume and an Applied Biosystems StepOnePlus™ instrument. 2 µl of DNA were used for each sample to a final volume of 15 µl. The final concentration of each primer was 0.5 µM. The amount of DNA was normalized using the difference in threshold cycle (CT) values (ΔCT) between rpL32 and target genes.

The quantitative real-time PCR (qPCR) primer sequences for *Drosophila melanogaster* ribosomal protein L32 gene (*RpL32*) were 5′-GCTAAGCTGTCGCAACAAAT-3′ (forward) and 5′-GAACTTCTTGAATCCGGTGGG-3′ (reverse).

Sequences for *Parg* were 5′-AGAAACACCCTCAAGAGGAAG-3′ (forward) and 5′-CGCTCTGTGGGACACAC-3′ (reverse).

Sequences for *Cg14850* were 5′-GCCTATTGAGGAGTAGCGAG-3′ (forward) and 5′-TGTCGTGGTATCTGTTCCATC-3′ (reverse).

Sequences for *Cyp6w1* were 5′-TTACATCTGGCAAGATCAAGC-3′ (forward) and 5′-TCACTTGGACTTCCGTACC-3′ (reverse).

Sequences for *Ninad* were 5′-GCCCCACATTTACCTTCATTG-3′ (forward) and 5′-AGAGATGTCCACCATTCGC-3′ (reverse).

Sequences for *Nfat* were 5′-AAAGACAGCCGGGTAAGGGAT-3′ (forward) and 5′-CAGGAACCATTTTGCCAGGAC-3′ (reverse).

Sequences for *Aest7* were 5′-AACCTCGGCTTTGTGGAG-3′ (forward) and 5′-CTGAAGTAGGGCACATCGTAG-3′ (reverse).

Sequences for *Mtnc* were 5′-GCTGCGGAACAAACTGC-3′ (forward) and 5′-GCCATTCTTGCACACGC-3′ (reverse).

### GO-terms overrepresentation among DEGs

GO-terms overrepresentation among PARG and PARP-1 DEGs was performed using the STRING database of known and predicted protein–protein interactions^25^. Upregulated and downregulated DEGs were analyzed separately with the following settings: Network type—full STRING network, Required score—medium confidence (0.400), FDR stringency—medium (5 percent). PARP-1 list was based on *Parp-1* mutant microarray results we previously published^23^.

Developmental changes in the expression profile were determined using Graveley et al. data^24^ by comparing expression profiles between the two last third instar larvae stages: L3 larva puff stage 3–6 and L3 larva puff stage 7–9. A gene was considered significantly downregulated if its fold change was lower than -2 (hereinafter termed as developmental downregulated DEGs), while it was considered significantly upregulated if its fold change was higher than 2 (hereinafter termed as developmental upregulated DEGs). Among the 215 PARG downregulated DEGs, 96 were developmental upregulated DEGs, while among the 797 PARG upregulated DEGs, 416 were developmental downregulated DEGs. Among the 311 PARP-1 downregulated DEGs, 81 were developmental upregulated DEGs, while among the 392 PARP-1 upregulated DEGs, 172 were developmental downregulated DEGs. Finally, among the 51 PARG/PARP-1 common downregulated DEGs, 29 were developmental upregulated DEGs, while among the 234 PARG/PARP-1 common upregulated DEGs, 151 were developmental downregulated DEGs.

### Motifs analysis

Promoter sequences were extracted using the Eukaryotic Promoter Database (EPD)^53^. Only the most representative promoter was selected for each gene. The promoter sequences included 500 base pairs upstream from the TSS and 100 base pairs downstream. Motif analysis was performed using Sensitive, Thorough, Rapid, Enriched Motif Elicitation (STREME)^54^ with the default settings. Control promoter sequence lists were generated by randomly choosing the same number of genes as that found in either the downregulated or upregulated DEG lists. 5 different control lists were generated, they all display identical results when compared to the group lists. The motifs found with STREME were compared to the TomTom database^42^ with the default settings.

## Supplementary Information

Supplementary Information.

## Data Availability

Mutant strains and transgenic stocks are available upon request. The authors state that all data necessary to confirm the conclusions presented in the article are represented fully within the article. RNA-seq raw data are accessible upon demand or on GEO platform with this link: https://www.ncbi.nlm.nih.gov/geo/query/acc.cgi?acc=GSE200499.
