# Supplementary material for: Poly(ADP-ribosyl)ating enzymes cooperate to coordinate development
Source: Sci Rep. 2022 Dec 21;12:22120. doi: 10.1038/s41598-022-26530-2 (PMC9772176; doi:10.1038/s41598-022-26530-2)
Supplement: Supplementary file 1 — Supplementary Information. [file 41598_2022_26530_MOESM1_ESM.pdf]

SUPPLEMENTARY APPENDIX FOR

Poly(ADP-ribosyl)ating enzymes cooperate to coordinate development

Guillaume Bordet, Yaroslava Karpova and Alexei V. Tulin

University of North Dakota, Grand Forks, ND

Address correspondence to:

E-mail: Alexei.Tulin@und.edu

THIS FILE INCLUDES:

Supplemental Figure S1. *Parp-1* and *Parg* expression profile.

Supplemental Figure S2. qPCR confirmation results correlate with RNA-seq results.

Supplemental Figure S3. The co-localization of PARG-EGFP and PARP-1-DsRed proteins in *Drosophila* polytene chromosomes.

Supplemental Figure S4. Expression profile of 66 PARG upregulated DEGs involved in proteolysis that should be downregulated during development**.**

Supplemental Figure S5. Functions of the PARG and PARP-1 DEGs involved in oxido-reduction process.

Supplemental Figure S6. A GATA motif is enriched at the promoter region of PARG and PARP-1 upregulated DEGs

Supplemental Figure S7. GATA factors undergo an increase in expression before L3 larva puff stage 7-9.

Supplemental Table S1. PARG DEGs involved in defense response are downregulated.

Supplemental Table S2. PARG DEGs involved in cuticle formation are downregulated at the end of third instar larvae.

Supplemental Table S3. PARG DEGs involved in proteolysis are mainly digestive enzymes.

Supplemental Table S4. PARG DEGs are organized in clusters of genes sharing the same functions.

Supplemental Table S5. The expression of ecdysone early-response genes is not affected in Parg or Parp-1 mutants.

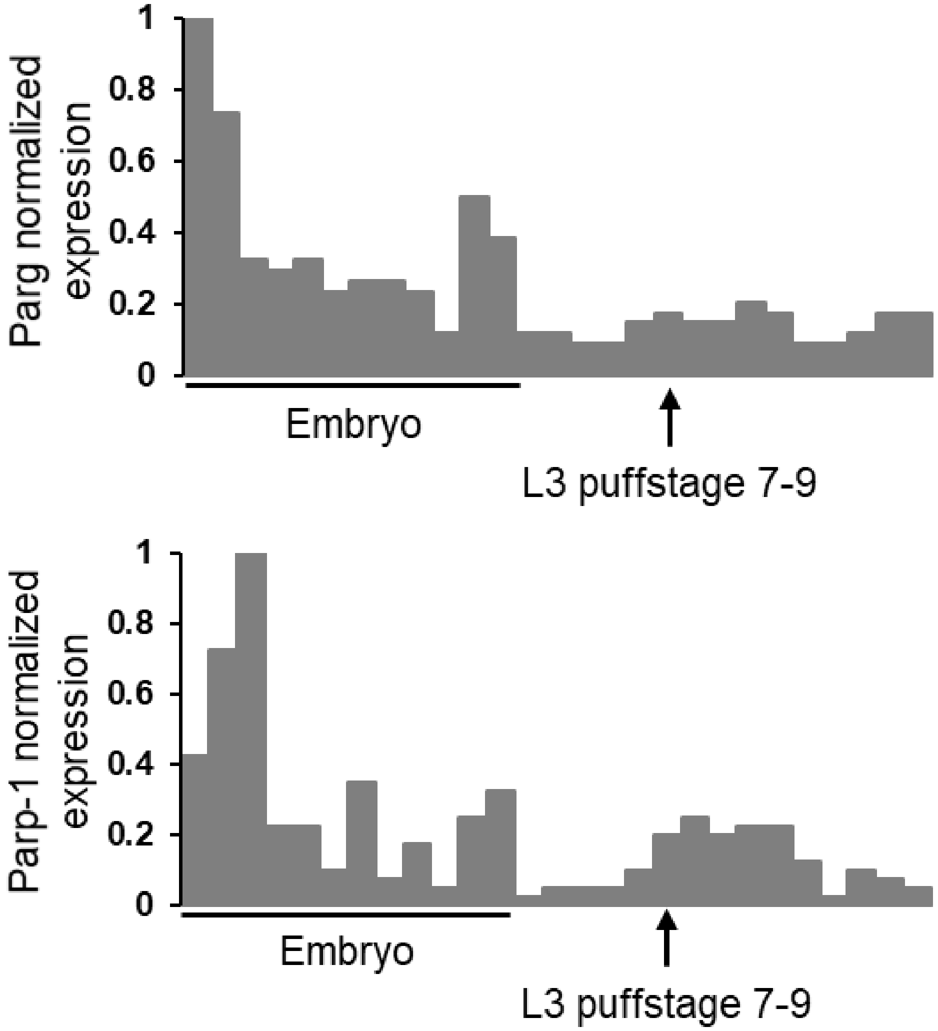

Figure S1: *Parp-1 and Parg expression profile*. *Parg* (upper panel) and *Parp-1* (lower panel) expression profile from early embryo to adult. The expression is normalized to their maximum expression. The black bar corresponds to embryonic development, while the arrow indicates stage L3 puff stage 7-9, which is the last stage before the death of *Parg* and *Parp-1* mutants. These graphs were constructed using RNA-seq data published in Graveley et al. (Graveley et al., 2011).

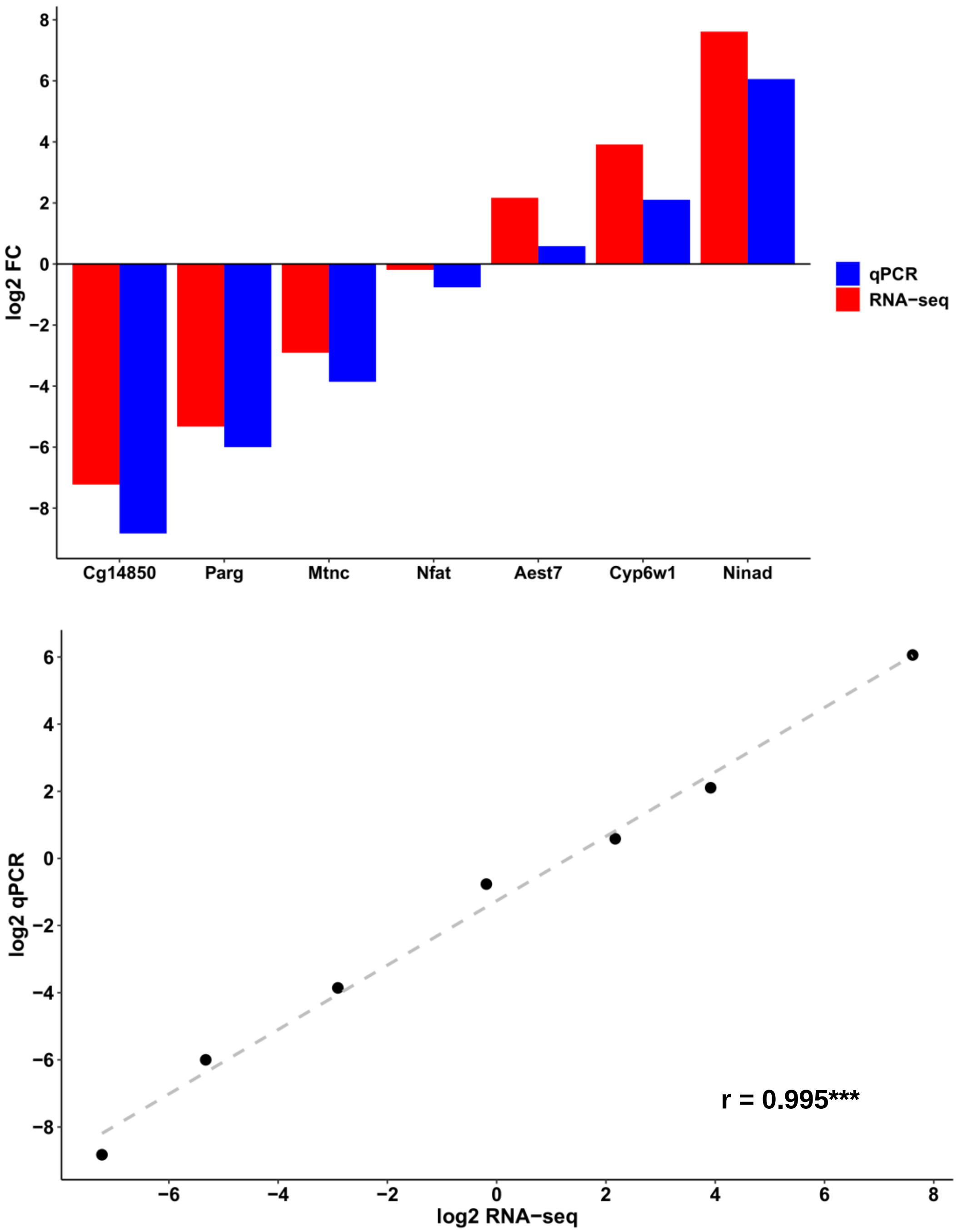

Figure S2: ***qPCR confirmation results correlate with RNA-seq results***. The upper panel represents the log2 scale fold change (FC) of seven target genes in the absence of PARG compared to control. qPCR results are represented in blue, while RNA-seq results are represented in red. The lower panel is a scatter plot of the log2 scale FC observed in RNA-seq plotted on the X-axis and the log2 scale of FC observed during qPCR confirmation plotted on the Y-axis. The r number is the Pearson correlation coefficient. The *p*-value is calculated based on the Pearson correlation coefficient and the *t*-distribution with n-2 degrees of freedom. ***: *p*-value < 0.01.

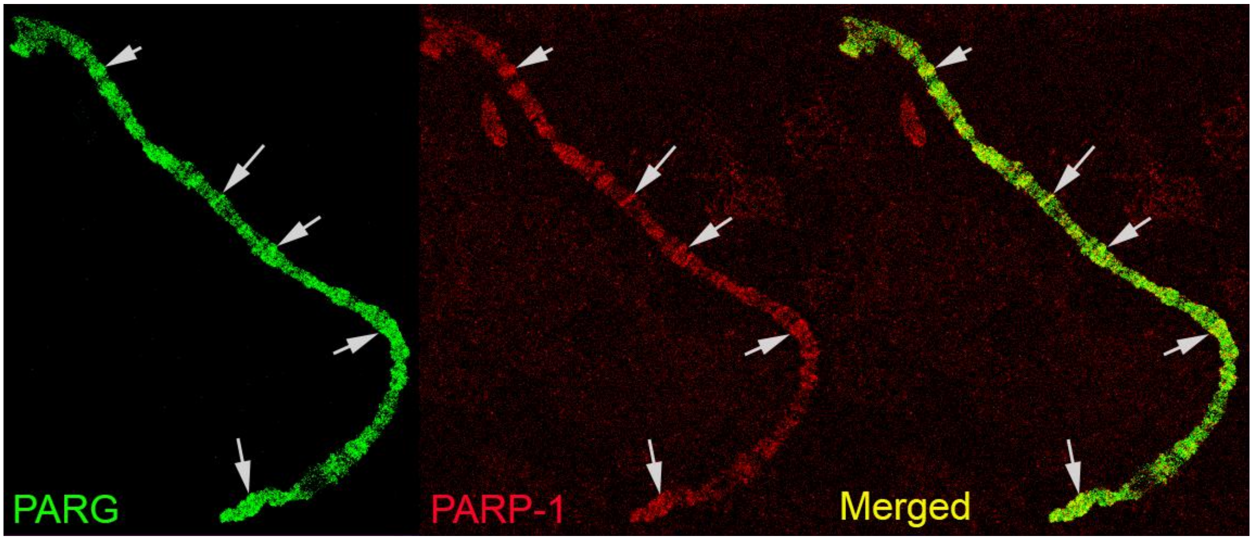

Figure S3: The co-localization of PARG-EGFP and PARP-1-DsRed proteins in *Drosophila* polytene chromosomes of the *Parg*$^{27.1}$ line were immune-stained with the anti-GFP and anti-RFP antibody. DNA staining is not included due to severe crosstalk of DNA dyes with the red channel masking the specific staining of the PARP-1-DsRed. : Arrows indicate sites with the severe proteins co-localization. The image was obtained using the Leica SPE confocal microscope.

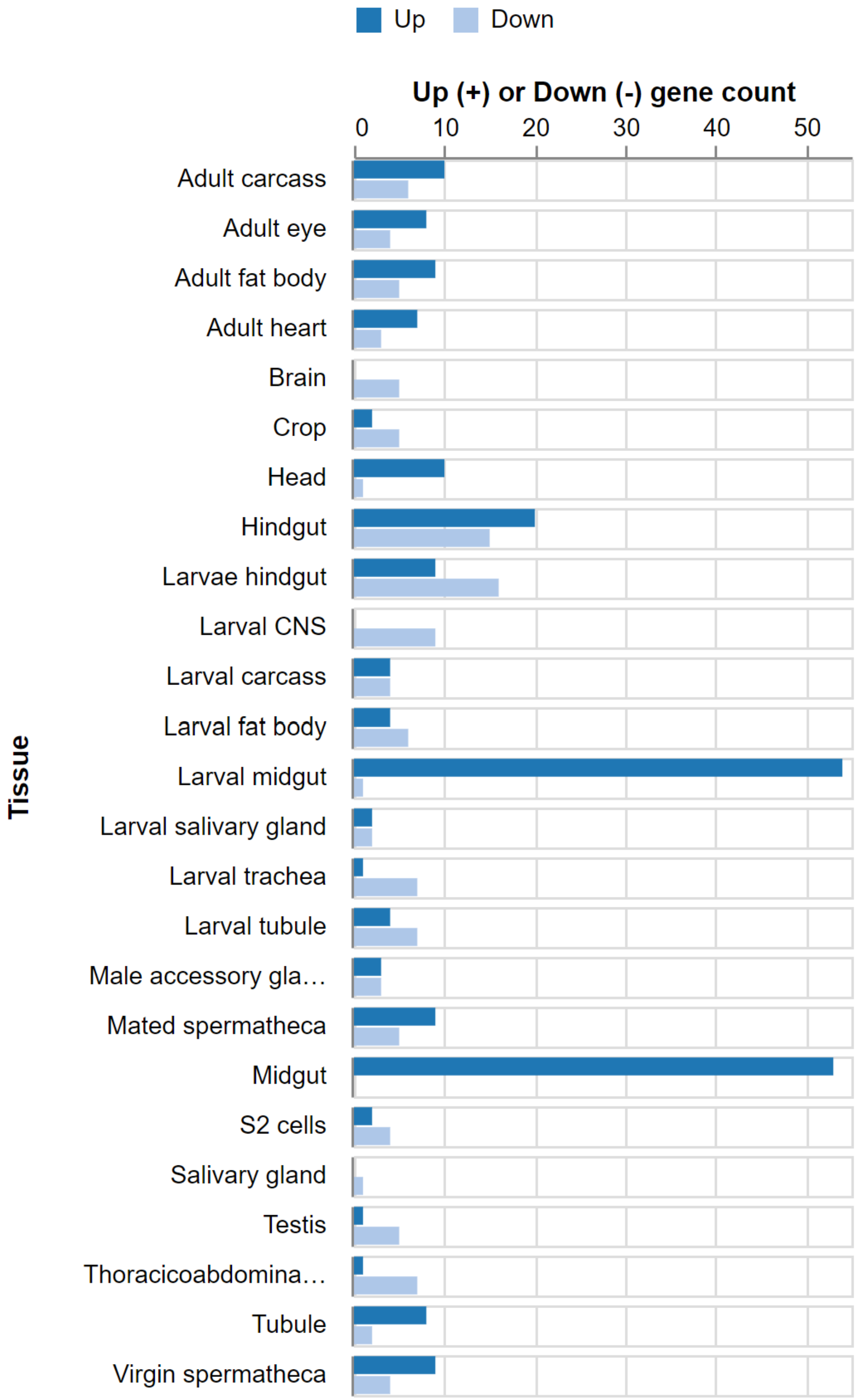

Figure S4: ***Expression profile of 66 PARG upregulated DEGs involved in proteolysis that should be downregulated during development.*** This graph shows 66 genes that present a higher expression than average for each tissue. The 'Up' category highlighted in dark blue corresponds to the genes that present a higher expression than average, while the 'Down' category highlighted in light blue corresponds to genes that present a lower expression than average. The expression of 53 of these genes (80.3%) is enriched in midgut tissue. This graph was obtained using the Flymine tool (Lyne et al., 2007).

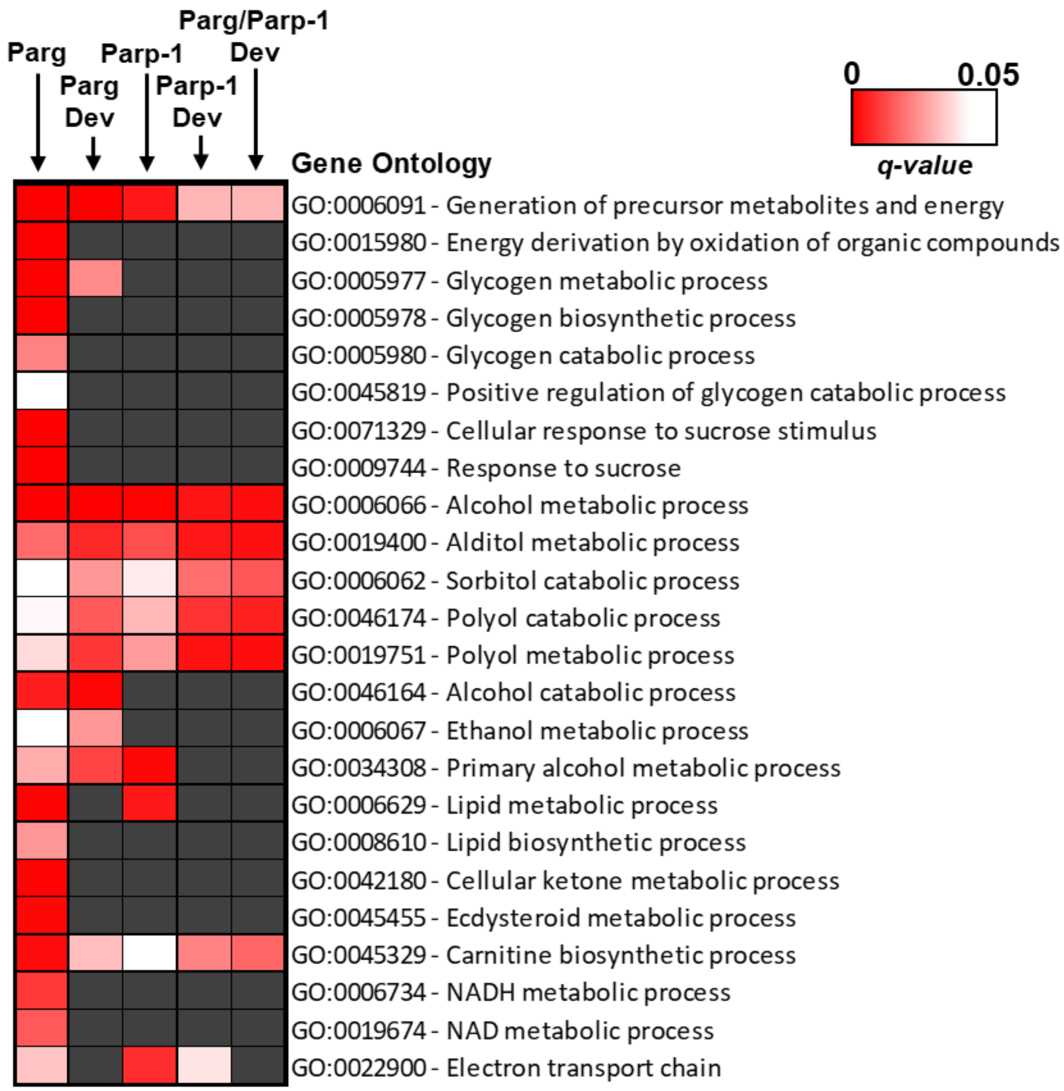

Figure S5: **Functions of the *PARG and PARP-1 DEGs involved in oxido-reduction process.*** Overview of the main Gene Ontology-terms (GO-terms) that are overrepresented among the upregulated DEGs involved in oxido-reduction. The left part corresponds to a heatmap of the FDR-corrected *p*-values (*q*-value) associated with different GO-terms. The red-to-white shaded tiles correspond to GO-terms significantly overrepresented among DEGs, while dark gray tiles correspond to GO-terms not significantly enriched. The left column, titled “Parg”, includes 103 Parg upregulated DEGS involved in oxido-reduction. The second column, titled “Parg Dev”, is a subtype of the first column that includes the 52 PARG upregulated DEGs involved in oxido-reduction and should be downregulated through development. The third column, titled “Parp-1”, includes the 66 PARP-1 upregulated DEGS involved in oxido-reduction. The fourth column, titled “Parp-1 Dev”, is a subtype of the third column that includes the 30 PARP-1 upregulated DEGs involved in oxido-reduction and should be downregulated through development. The fifth column, titled “Parg/Parp-1 Dev”, includes the 26 PARG/PARP-1 common upregulated DEGs involved in oxido-reduction that should be downregulated through development. *q*-value is a Benjamini–Hochberg corrected *p*-value based on multiple tests performed by STRING (Szklarczyk et al., 2021). The heatmap was generated using the Excel 2016 software, available at this link: https://www.microsoft.com/en-us/microsoft-365/excel.

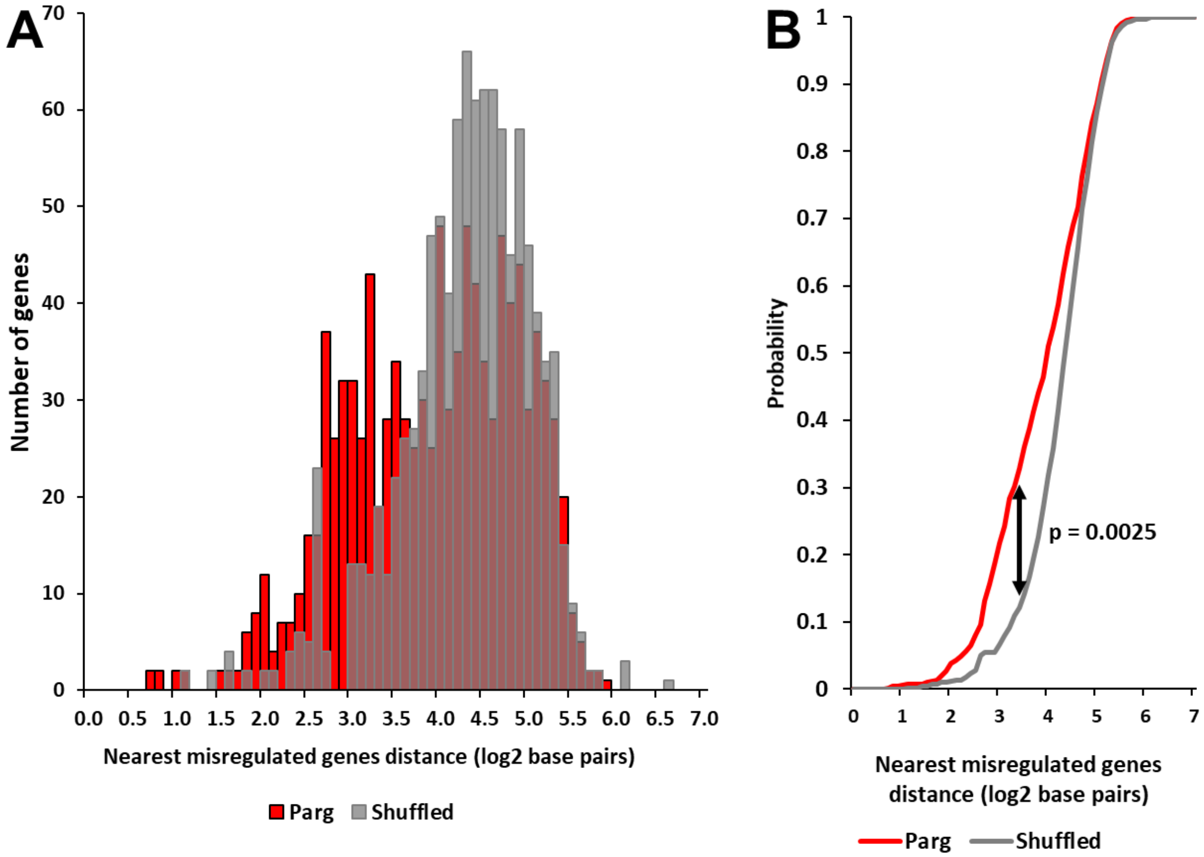

Figure S6: ***PARG DEGs are closer together than expected given that DEG positions are random.*** A) Distribution of the distance (in log2 base pairs) between each PARG DEG and the nearest DEG (red) or between genes chosen randomly from a list of 1012 genes (gray). B) Cumulative distribution of the data presented in panel A. The difference between the two distributions was investigated using a Kolmogorov-Smirnov test (Massey, 1951).

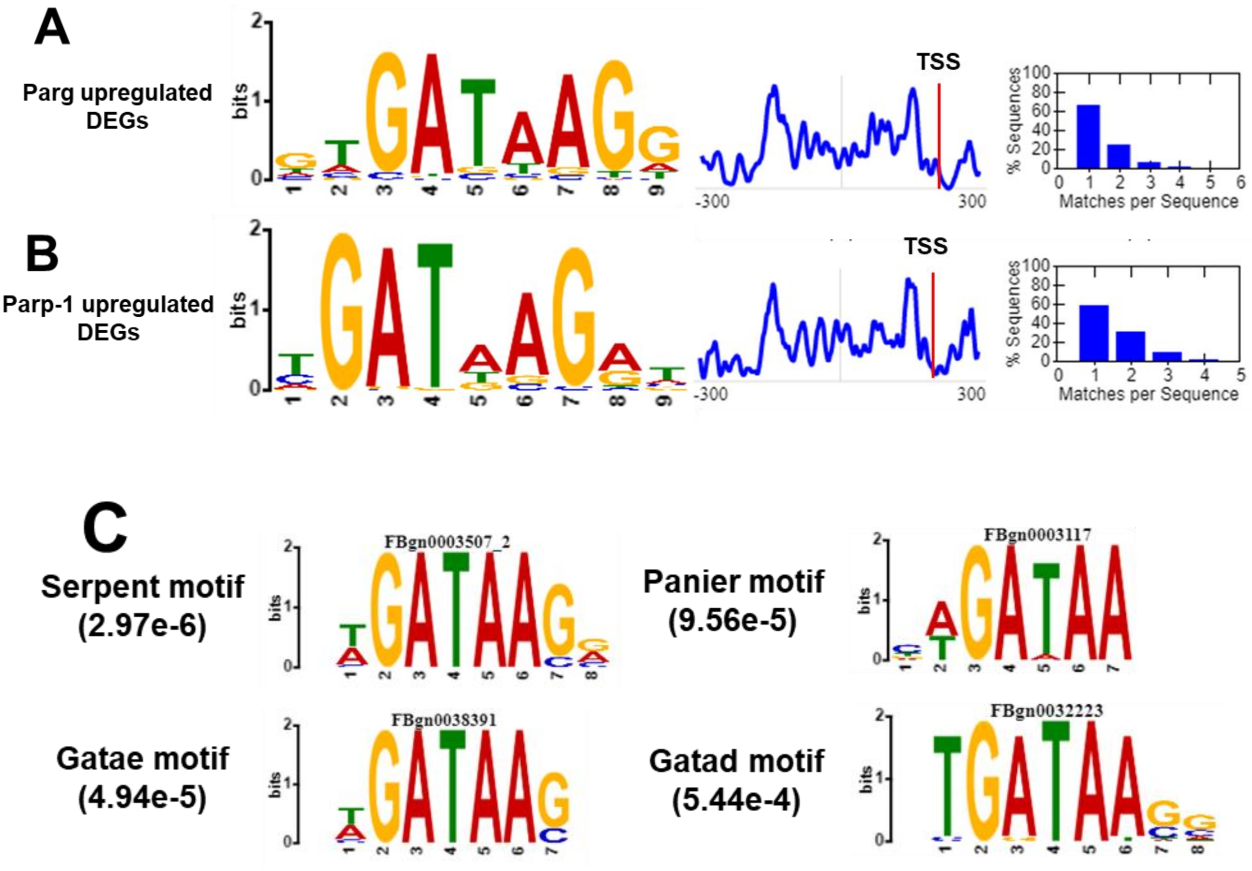

Figure S7: ***A GATA motif is enriched at the promoter region of PARG and PARP-1 upregulated DEGs.*** **A-B)** Left panels indicate the top motif found by STREME among the promoter regions of **A)** PARG upregulated DEGs and **B)** PARP-1 upregulated DEGs. The promoter sequences of DEGs were compared to promoter sequences of genes chosen randomly. The middle panels show the distribution of the motifs presented on the left panels around the TSS. The TSS is highlighted with a red line. The two major sites where this motif is found are around -400 and -50 base pairs from the TSS. The right panels indicate the occurrence per sequence of the motifs presented in the left panels. 65% of PARG upregulated DEGs positive for this motif have it in a single copy and around 58% for PARP-1. **C)** Comparison of the motif sequence presented in panel **A** with known motifs using TOMTOM tools. The top four motifs found are four GATA factors. The *p*-value of each motif is indicated in brackets. The motif of Grain, the last *Drosophila* GATA factor, is not found among significant motifs.

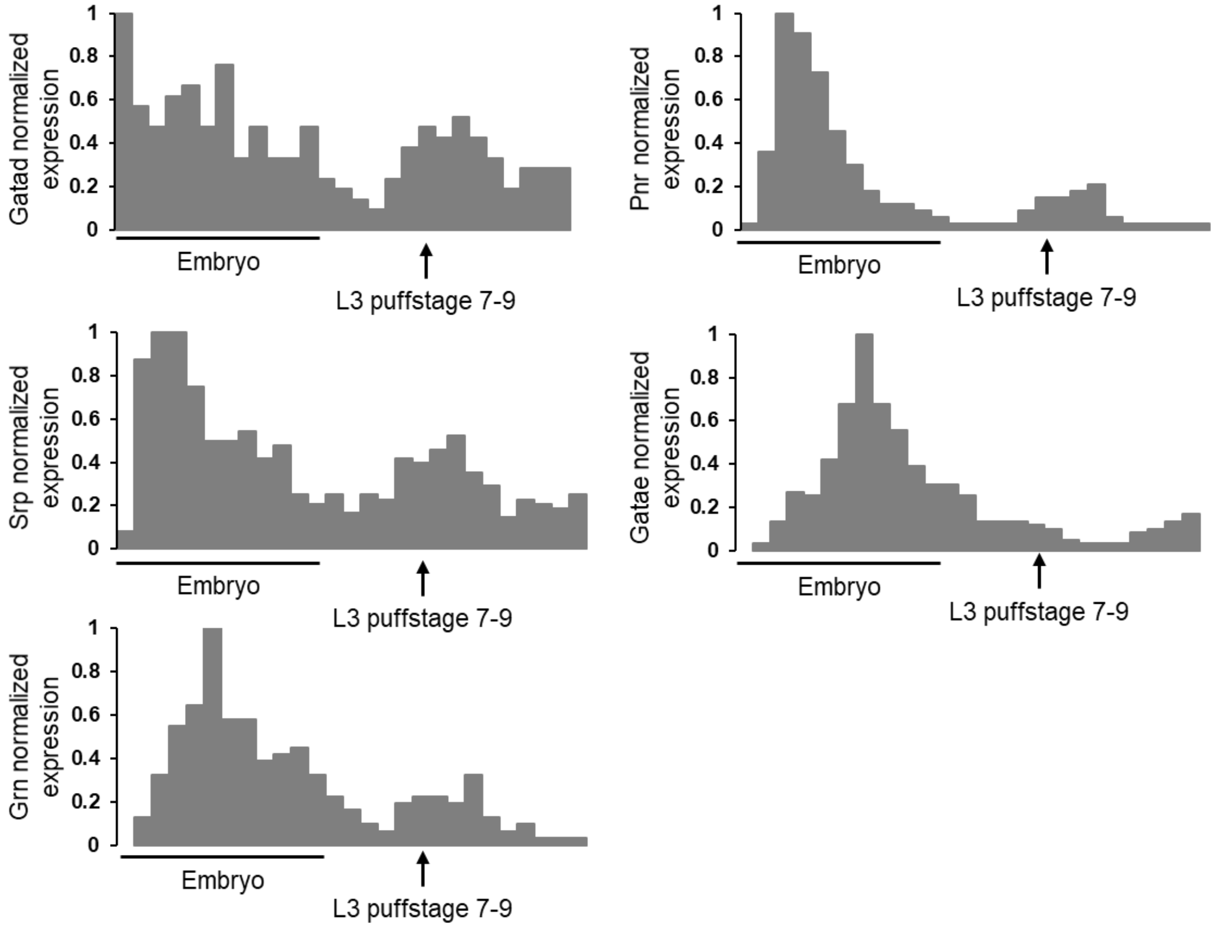

Figure S8: ***GATA factors undergo an increase in expression before L3 larva puff stage 7-9***. Expression profile of GATA factors from early embryo to adult. The expression is normalized to maximum. The black bar corresponds to embryonic development, while the arrow indicates stage L3 puff stage 7-9, the last stage before the death of *Parg* and *Parp-1* mutants. These graphs were construced using RNA-seq data published in Graveley et al. (Graveley et al., 2011).

Gene Name	Flybase ID	PARG FC
CG32706	FBgn0052706	-2.22
insc	FBgn0011674	-2.56
Drsl2	FBgn0052279	-3.77
Drsl5	FBgn0035434	-7.43
Drs	FBgn0283461	-3.04
Eig71Eg	FBgn0004594	-4.86
PGRP-SD	FBgn0035806	-2.30
slif	FBgn0037203	-2.76
Tep1	FBgn0041183	-3.72
SPH93	FBgn0032638	-3.01
Victoria	FBgn0053117	-22.77
Sid	FBgn0039593	-2.56
GNBP-like3	FBgn0034511	-5.97
DptA	FBgn0004240	-4.30
IM23	FBgn0034328	-2.83
CG6429	FBgn0046999	-3.90
Mtk	FBgn0014865	-8.23
Listericin	FBgn0033593	-3.71
JhI-21	FBgn0028425	-2.13
NT1	FBgn0261526	-3.04
Dro	FBgn0010388	-9.33
PGRP-SB2	FBgn0043577	-15.68
CG32706	FBgn0052706	-2.22
CG9518	FBgn0030590	-6.69
Pvf1	FBgn0030964	-2.23
cyc	FBgn0023094	-2.21
rpr	FBgn0011706	-2.79
Hsp67Ba	FBgn0001227	-2.28
Hsp67Bc	FBgn0001229	-4.14
Ho	FBgn0037933	-2.26
Hsp70Bb	FBgn0013278	-1.94
Cht5	FBgn0038180	-3.27
Gbp1	FBgn0034199	-2.87
CG12896	FBgn0033521	-3.41
Mmp2	FBgn0033438	-2.29
phr	FBgn0003082	-3.36
Gyc89Da	FBgn0038435	-3.65
Eip93F	FBgn0264490	-3.41

Table S1: *PARG DEGs involved in defense response are downregulated*. List of 38 PARG DEGs that are downregulated in *Parg* mutant and are all involved in defense response function. FC: Fold change between control and *Parg* mutant groups.

				No expression			High expression		
Name	Larva L1	Larva L2	Larva L3 12hr	Larva L3 puff stage 1-2	Larva L3 puff stage 3-6	Larva L3 puff stage 7-9	White prepupa	prepupa 12hr	PARG FC
Cht9	71	184	145	30	4	2	2	3	6.006201
Cpr60D	12	13	507	318	90	15	2	5	8.417363
Lcp9	0	0	882	1483	299	41	2	1	8.744008
Cpr11A	225	128	207	46	9	2	1	0	13.77179
Cpr11B	53	6	28	36	11	3	2	0	10.69907
TwdlX	71	121	202	62	12	4	0	1	8.793472
Cpr67Fa1	185	236	4005	337	35	6	1	5	103.7984
Cpr67Fa2	195	139	2971	199	36	12	2	5	7.361233
Lcp65Ac	305	60	1015	194	68	22	1	23	4.656438
Lcp65Aa	60	7	127	65	6	1	0	1	36.26479
Cpr65Aw	1	2	14	93	18	1	0	0	9.287663
Cpr65Av	25	62	173	104	21	3	0	6	4.106832
Lcp65Ae	1	8	1025	15	1	0	0	4	106.6872
Lcp65Af	32	197	2053	9	2	0	0	8	6.301471
Lcp65Ag2	874	201	702	10	3	1	0	593	292.2678
Cpr65Au	0	1	1	10	2	0	0	2	13.95029
Lcp65Ag3	1331	236	825	36	5	1	0	11	485.8648
l(3)mbn	7	1	67	74	27	4	0	0	5.327119
FASN2	2	5	8	5	2	0	0	0	7.616712
Cpr78Cc	383	134	695	210	18	1	3	750	70.82021
obst-E	190	66	351	123	36	8	1	27	7.015062
Ccp84Ag	7	106	562	26	3	0	0	314	4.88004
Cpr100A	45	15	80	16	9	3	2	4150	4.093107
Cpr49Af	4	1	124	198	24	4	0	9	16.52748
Cpr47Eb	95	357	138	45	11	3	3	33	5.984466
Cpr47Eg	3707	362	9561	610	14	3	1	2	1320.862
Cpr47Ec	7	61	595	216	20	1	0	78	68.39212
Lcp4	74	35	4350	13696	7378	2128	91	160	4.04368
CG15754	0	0	20	7	2	0	0	0	12.76691
CG32564	997	287	392	37	5	1	0	6	59.62545
CG15515	451	60	944	139	8	1	0	3	167.6079

Table S2: ***PARG DEGs involved in cuticle formation are downregulated at the end of third instar larvae***. List of 31 PARG upregulated DEGs that should be downregulated during development and are involved in cuticle formation. The red-shade colors correspond to the reported expression of the genes at a specific stage (Graveley et al., 2011), ranging from high expression (red) to no expression (white). The stages displayed in the picture start from Larva L1 to prepupa 12hr. The last column shows the Fold change (FC) observed during L3 larva puff stage 7-9 in the absence of PARG. The expression of these 31 genes decreases at the end of third instar larvae, becoming almost nil during white prepupa state for 30 of them.

Flybase ID	Symbol	Gene Ontology	PARG Fold Change	Development Fold Change
FBgn0051343	CG31343	Aminopeptidases	33.2	-4.2
FBgn0051233	CG31233	Aminopeptidases	8.8	-5.4
FBgn0051198	CG31198	Aminopeptidases	10.9	-4.4
FBgn0038136	CG8774	Aminopeptidases	120.1	-8.0
FBgn0038135	CG8773	Aminopeptidases	8.3	-4.2
FBgn0052483	CG32483	Carboxypeptidases	14.0	-4.3
FBgn0035718	CG14820	Carboxypeptidases	16.9	-4.0
FBgn0035154	CG3344	Carboxypeptidases	2.6	-2.3
FBgn0032144	f	Carboxypeptidases	2.9	-3.1
FBgn0031930	CG7025	Carboxypeptidases	27.6	-3.9
FBgn0031929	CG18585	Carboxypeptidases	5.5	-4.1
FBgn0033659	Damm	Cysteine type endopeptidases	4.7	-2.4
FBgn0028950	CG15255	Metallopeptidases	8.4	-7.6
FBgn0028949	CG15254	Metallopeptidases	38.1	-7.2
FBgn0028948	CG15253	Metallopeptidases	62.8	-7.7
FBgn0028945	CG7631	Metallopeptidases	35.1	-7.0
FBgn0263234	Phae1	Serine type endopeptidases	2.2	-2.0
FBgn0053127	CG33127	Serine type endopeptidases	10.1	-3.9
FBgn0051269	CG31269	Serine type endopeptidases	12.7	-3.1
FBgn0051267	CG31267	Serine type endopeptidases	9.1	-7.7
FBgn0051266	CG31266	Serine type endopeptidases	11.9	-4.0
FBgn0051265	CG31265	Serine type endopeptidases	75.9	-9.4
FBgn0043471	κTry	Serine type endopeptidases	15.6	-6.5
FBgn0043470	λTry	Serine type endopeptidases	262.3	-6.3
FBgn0040060	yip7	Serine type endopeptidases	4.7	-2.4
FBgn0038484	CG5246	Serine type endopeptidases	3.2	-3.0
FBgn0038482	CG4053	Serine type endopeptidases	6.8	-3.4
FBgn0038481	CG17475	Serine type endopeptidases	16.9	-8.2
FBgn0038479	CG17477	Serine type endopeptidases	6.1	-3.0
FBgn0036024	CG18180	Serine type endopeptidases	3.6	-3.4
FBgn0035887	Jon66Cii	Serine type endopeptidases	4.2	-4.9
FBgn0035886	Jon66Ci	Serine type endopeptidases	3.7	-3.8
FBgn0035670	CG10472	Serine type endopeptidases	20.0	-4.9
FBgn0035665	Jon65Aiii	Serine type endopeptidases	4.3	-2.0
FBgn0034052	CG8299	Serine type endopeptidases	3.5	-2.5
FBgn0031249	CG11911	Serine type endopeptidases	10.7	-3.9
FBgn0030776	CG4653	Serine type endopeptidases	4.3	-3.2
FBgn0030775	CG9673	Serine type endopeptidases	4.5	-3.9
FBgn0030774	spheroide	Serine type endopeptidases	7.9	-3.3
FBgn0030688	CG8952	Serine type endopeptidases	2.9	-2.3
FBgn0015001	ıTry	Serine type endopeptidases	2.2	-3.1
FBgn0011834	Ser6	Serine type endopeptidases	34.7	-4.0
FBgn0011556	ζTry	Serine type endopeptidases	6.5	-3.8
FBgn0011555	θTry	Serine type endopeptidases	8.9	-2.6
FBgn0010425	εTry	Serine type endopeptidases	6.0	-2.3
FBgn0010357	βTry	Serine type endopeptidases	6.7	-2.5
FBgn0003358	Jon99Ci	Serine type endopeptidases	2.9	-2.1

Table S3: *PARG DEGs involved in proteolysis are mainly digestive enzymes*. List of 47 PARG upregulated DEGs that should be downregulated during development and are reported as digestive enzymes. The first column indicates the Flybase ID of the genes, while the second column indicates the gene name. The third column indicates the molecular function of the genes. The fourth column indicates the fold change observed between *Parg* mutant and control groups, while the fifth column indicates the fold change between L3 larva puff stage 3-6 and 7-9 stage.

Cluster	Name	Function	PARG FC	Cluster	Name	Function	PARG FC
3L (1)	l(3)mbn	Chitin constituent	5.3	3L (6)	CG9119	Hydrolase	3.9
	Lcp65Ag3	Chitin constituent	485.9		LysB	Lyzozyme	4.2
	Cpr65Au	Chitin constituent	14.0		LysD	Lyzozyme	5.1
	Lcp65Ag2	Chitin constituent	292.3		LysE	Lyzozyme	6.0
	Lcp65Ag1	Chitin constituent	423.8		LysP	Lyzozyme	-3.1
	Lcp65Af	Chitin constituent	6.3		LysS	Lyzozyme	3.0
	Lcp65Ae	Chitin constituent	106.7		CG33965	Phosphatidylinositol biphosphate binding	5.6
	Cpr65Av	Chitin constituent	4.1		CG33966	Phosphatidylinositol biphosphate binding	4.2
	Cpr65Aw	Chitin constituent	9.3		CG9194	Two-pore domain K+ channel subunit	3.8
	Lcp65Ad	Chitin constituent	2.6	2R (1)	lambdaTry	Trypsin-like	262.3
	Lcp65Ac	Chitin constituent	4.7		kappaTry	S1A non-peptidase	15.6
	Lcp65Ab2	Chitin constituent	2.2		zetaTry	Tryspin	6.5
	Cpr65Ax1	Chitin constituent	208.8		etaTry	Tryspin	2.0
	Lcp65Ab1	Chitin constituent	11.2		thetaTry	Tryspin	8.9
	Cpr65Ax2	Chitin constituent	252.0		alphaTry	S1A serine protease	2.2
	Lcp65Aa	Chitin constituent	36.3		epsilonTry	S1A serine protease	6.0
	Acp65Aa	Chitin constituent	15.5		betaTry	Trypsin-like	6.7
	CG13297	Chitin constituent	3.9		gammaTry	Trypsin-like	6.2
	Cpr65Ay	Chitin constituent	12.9		CG30031	Trypsin-like	4.7
3L (2)	CG33986	Chitin binding	6.3		CG30025	Trypsin-like	5.8
	CG33985	Chitin binding	2.2		deltaTry	Trypsin-like	3.4
	CG42729	Chitin binding	3.0		CG13202	left-right symmetry	-1.0
	CG42728	Chitin binding	2.4		iotaTry	Trypsin-like	2.2
	obst-H	Chitin binding	2.6	2R (2)	Lcp4	Chitin constituent	4.0
3L (2)	CG5883	Chitin binding	7.8		Cyp4ad1	Other cytochrome P450	3.0
	CG7252	Chitin binding	3.5		Cyp4e2	Other cytochrome P450	1.8
	CG17826	Chitin binding	4.2		Cyp4e1	Other cytochrome P450	6.9
	obst-G	Chitin binding	4.0		Mal-A1	Alpha-glucosidase	2.6
	CG7248	Chitin binding	1.8		Mal-A2	Alpha-glucosidase	4.9
3L (4)	Cpr65Ax1	Chitin constituent	208.8		Mal-A3	Alpha-glucosidase	23.9
	Lcp65Ab1	Chitin constituent	11.2		Mal-A4	Alpha-glucosidase	3.8
	Cpr65Ax2	Chitin constituent	252.0		Mal-A5	Alpha-glucosidase	-1.0
	Lcp65Aa	Chitin constituent	36.3		Mal-A6	Alpha-glucosidase	1.0
	Acp65Aa	Chitin constituent	15.5		Mal-A7	Alpha-glucosidase	2.5
3L (5)	CG8563	Metallocarboxypeptidase	6.0		Mal-A8	Alpha-glucosidase	2.2
	CG32379	Metallocarboxypeptidase	-167.0	2L (1)	LManIII	Lysosomal mannosidase	9.9
	CG8562	Metallocarboxypeptidase	12.4		LManIV	Lysosomal mannosidase	13.3
	CG18417	Metallocarboxypeptidase	3.3		LManV	Lysosomal mannosidase	64.7
	CG8560	Metallocarboxypeptidase	2.4		LManVI	Lysosomal mannosidase	27.3

Table S4: PARG DEGs are organized in clusters of genes sharing the same functions. Example of clusters found among PARG DEGs. First and fifth columns indicate the chromosomes where the cluster is found. Second and sixth columns indicate the gene name. Third and seventh columns indicate the function of the genes. Fourth and eighth columns indicate the fold change observed between *Parg* mutant and control groups. Downregulated DEGs are highlighted in red.

Name	Function	Ctrl Av RPKM	PARG Av RPKM	FC
GstE14	Ecdysone synthesis	0.90	1.05	1.17
Nvd	Ecdysone synthesis	2.31	2.13	-1.08
Spok	Ecdysone synthesis	6.46	8.24	1.28
Sro	Ecdysone synthesis	24.15	15.04	-1.61
Cyp6t3	Ecdysone synthesis	0.39	0.44	1.11
Phm	Ecdysone synthesis	61.20	47.86	-1.28
Dib	Ecdysone synthesis	0.54	0.35	-1.56
Sad	Ecdysone synthesis	10.71	8.57	-1.25
Shd	Ecdysone synthesis	17.38	22.62	1.30
Usp	Ecdysone receptor (Early Response)	25.58	18.82	-1.36
Ecr	Ecdysone receptor (Early Response)	17.88	15.44	-1.16
Sgs1	Glue genes (early response)	82.68	83.72	1.01
Sgs8	Glue genes (early response)	4052.15	2450.64	-1.65
Sgs7	Glue genes (early response)	9731.65	6989.47	-1.39
Sgs3	Glue genes (early response)	4177.48	3061.10	-1.36
Sgs5	Glue genes (early response)	3835.17	2855.00	-1.34
Sgs4	Glue genes (early response)	2011.85	1376.18	-1.46
Eip74EF	Ecdysone response genes	31.11	23.81	-1.31
Eip75B	Ecdysone response genes	24.39	16.35	-1.49
Eip78C	Ecdysone response genes	35.79	12.96	-2.76
Hr3	Ecdysone response genes	11.36	3.33	-3.41
Hr4	Ecdysone response genes	13.39	7.00	-1.91
Hr39	Ecdysone response genes	22.04	15.40	-1.43
Br	Ecdysone response genes	54.95	38.41	-1.43

Table S5: The expression of ecdysone early-response genes is not affected in *Parg* or *Parp-1* mutants.

Expression profile enzymes responsible of the synthesis of 20-hydroxyecdysone (top panel), main ecdysone receptors (middle top panel), ecdysone early-response genes (middle bottom panel)

and ecdysone response genes (bottom panel). Third and Fourth columns show the average Read per Kilobase Million (Av RPKM) observed between the three biological replicates in control and in *Parg* mutant respectively. Fifth column is fold change (FC) between control and *Parg* mutant. Genes that are significantly downregulated are highlighted in red.

REFERENCES

Graveley, B. R., Brooks, A. N., Carlson, J. W., Duff, M. O., Landolin, J. M., Yang, L., Artieri, C. G., van Baren, M. J., Boley, N., Booth, B. W., Brown, J. B., Cherbas, L., Davis, C. A., Dobin, A., Li, R., Lin, W., Malone, J. H., Mattiuzzo, N. R., Miller, D., . . . Celniker, S. E. (2011). The developmental transcriptome of Drosophila melanogaster. *Nature*, *471*(7339), 473-479. https://doi.org/10.1038/nature09715

Lyne, R., Smith, R., Rutherford, K., Wakeling, M., Varley, A., Guillier, F., Janssens, H., Ji, W., McLaren, P., North, P., Rana, D., Riley, T., Sullivan, J., Watkins, X., Woodbridge, M., Lilley, K., Russell, S., Ashburner, M., Mizuguchi, K., & Micklem, G. (2007). FlyMine: an integrated database for Drosophila and Anopheles genomics. *Genome Biol*, *8*(7), R129. https://doi.org/10.1186/gb-2007-8-7-r129

Massey, F. J. (1951). The Kolmogorov-Smirnov Test for Goodness of Fit. *Journal of the American Statistical Association*, *46*(253), 68-78. https://doi.org/10.1080/01621459.1951.10500769

Szklarczyk, D., Gable, A. L., Nastou, K. C., Lyon, D., Kirsch, R., Pyysalo, S., Doncheva, N. T., Legeay, M., Fang, T., Bork, P., Jensen, L. J., & von Mering, C. (2021). The STRING database in 2021: customizable protein-protein networks, and functional characterization of user-uploaded gene/measurement sets. *Nucleic Acids Res*, *49*(D1), D605-D612. https://doi.org/10.1093/nar/gkaa1074
